# Structure based screening and molecular docking with dynamic simulation of natural secondary metabolites to target RNA-dependent RNA polymerase of five different retroviruses

**DOI:** 10.1371/journal.pone.0307615

**Published:** 2024-08-05

**Authors:** Muhammad Azeem, Ghulam Mustafa, Sibtain Ahmed, Amna Mushtaq, Muhammad Arshad, Muhammad Usama, Muhammad Farooq

**Affiliations:** 1 College of Life Sciences, Anhui Normal University, Wuhu, China; 2 Department of Biochemistry, Government College University Faisalabad, Faisalabad, Pakistan; 3 Department of Biochemistry, Bahauddin Zakariya University, Multan, Pakistan; 4 Department of Medical Laboratory, TIMES Institute, Multan, Pakistan; 5 Department of Basic Sciences, University of Veterinary and Animal Sciences, Jhang-Campus, Lahore, Pakistan; Saveetha University - Poonamallee Campus: SIMATS Deemed University, INDIA

## Abstract

Viral diseases pose a serious global health threat due to their rapid transmission and widespread impact. The RNA-dependent RNA polymerase (RdRp) participates in the synthesis, transcription, and replication of viral RNA in host. The current study investigates the antiviral potential of secondary metabolites particularly those derived from bacteria, fungi, and plants to develop novel medicines. Using a virtual screening approach that combines molecular docking and molecular dynamics (MD) simulations, we aimed to discover compounds with strong interactions with RdRp of five different retroviruses. The top five compounds were selected for each viral RdRp based on their docking scores, binding patterns, molecular interactions, and drug-likeness properties. The molecular docking study uncovered several metabolites with antiviral activity against RdRp. For instance, cytochalasin Z8 had the lowest docking score of –8.9 (kcal/mol) against RdRp of SARS-CoV-2, aspulvinone D (–9.2 kcal/mol) against HIV-1, talaromyolide D (–9.9 kcal/mol) for hepatitis C, aspulvinone D (–9.9 kcal/mol) against Ebola and talaromyolide D also maintained the lowest docking score of –9.2 kcal/mol against RdRp enzyme of dengue virus. These compounds showed remarkable antiviral potential comparable to standard drug (remdesivir –7.4 kcal/mol) approved to target RdRp and possess no significant toxicity. The molecular dynamics simulation confirmed that the best selected ligands were firmly bound to their respective target proteins for a simulation time of 200 ns. The identified lead compounds possess distinctive pharmacological characteristics, making them potential candidates for repurposing as antiviral drugs against SARS-CoV-2. Further experimental evaluation and investigation are recommended to ascertain their efficacy and potential.

## Introduction

Everyone has gained a lot of knowledge about viruses after the Covid-19 outbreak over the past three years. The severe acute respiratory syndrome coronavirus 2 (SARS-CoV-2), human immunodeficiency virus (HIV), hepatitis C virus (HCV), dengue, and Ebola viruses, belong to Retroviridae family, having RNA genome between 7 and 11 kb in size, are notable causative agents of major human diseases [1, 2]. The enzyme known as reverse transcriptase, which was independently identified in 1971 by American virologists Howard Temin and David Baltimore, gave rise to the term of "retrovirus" [3, 4]. The dual genetic feature enables the virus to spread from cell to cell as packaged RNA while leaving a copy of the DNA in each infected cell’s chromosomes, where it can pass from one generation to the next [5]. Research on retroviruses has significantly influenced the fields of genetics, molecular biology, biotechnology, medicine, cellular growth control and carcinogenesis.

The novel SARS-CoV-2 along HIV, HCV, dengue, and Ebola viruses, are currently without a reliable cure. In order to prevent the disease from spreading further, efforts were made too quickly to diagnose the infected individuals and subsequently isolate them [4]. Authorized medications and therapies are under clinical trials, and secondary compounds extracted from microbes and medicinal plants are all being explored continuously to cure infections caused by these retroviruses [6]. Scientists from various countries are currently striving to develop a vaccine against these viruses, but developing vaccines for RNA viruses, is a very difficult task.

Traditional treatments are mostly composed of natural secondary metabolites which are extracted from plants and microbes [7]. They are an important repository of essential chemicals required for the invention of novel pharmaceutical drugs, however, their application has declined in the previous 25 years due to practical challenges in investigating them in molecular target specific experiments [8]. These technical hurdles to natural metabolites assessment are now being diminished as new advanced approaches are developed. The therapeutic value of a phytochemical is directly proportionate to the mechanism by which it binds to its cellular target [9]. It is the most logical and important step in the medication development process. In retrovirus therapy, there are several pharmacological targets to inhibit, including the viral envelope protein, RNA, reverse transcriptase, integrase, and main protease enzyme [10, 11].

A cost-effective, time-efficient, and advantageous practical screening strategy is *in silico* target identification for metabolites based lead molecules [12]. The two most often employed *in silico* methodologies are quantitative structure activity relationship (QSAR) and molecular docking [13], whereas the molecular docking is more beneficial and fast approach for the screening of target macromolecules (protein, DNA, and RNA) [14]. The method also helps to study the mechanisms of action of natural compounds with their target proteins, as well as facilitating the invention of novel derived medications. Ligand-protein docking has resulted in the development of several secondary metabolite-based medications [10, 15]. Computational tools and software play vital role in drug discovery by accelerating the virtual screening, provide detailed simulation of drug-target interaction and precise molecular modeling [16]. These tools and software assist to identify the biological target and their validation, predict pharmacokinetics and pharmacodynamics and helps to optimize the lead compound(s) [17].

The RNA-dependent RNA polymerase (RdRp) participates in the synthesis, transcription, and replication of viral RNA, control the synthesis of RNA using an RNA template. RdRp has been recognized as a possible target for the development of anti-SARS-CoV-2 and other viral therapeutics [18]. The antiviral drugs based on nucleotide analogues that target the outer components of the RNA virus’s reproduction and functioning machinery [10, 19, 20]. Recently, natural products from marine source were evaluated as an anti-ZIKV agent by targeting RdRp [21]. The current study was therefore aimed to inhibit viral transcription, replication, and maturation by targeting RdRp with plant, fungal and bacterial derived secondary metabolites. Furthermore, comparing multiple interactions between ligand-receptor complexes would aid researchers in locating more exact target sites in receptor proteins. The combined targeting of numerous viral proteins with active residues can reveal a single leading treatment candidate against multiple proteins, which can be of immense help in controlling the infections in a timely manner. It will open the way for the investigation of further possible medication candidates with high bioavailability and efficacy against infections caused by retroviruses.

## Materials and methods

### Ligand structure acquisition

Literature and secondary metabolite databases were explored for the collection of compounds with antiviral potential. The 3D structures of 200 antiviral secondary metabolites were retrieved from NCBI’s Entrez PubChem database (https://pubchem.ncbi.nlm.nih.gov/; accessed on 07 March 2023) in SDF format. The phytochemicals were energy minimized and then converted into pdbqt format through Open Babel tool [22] of PyRx. The 3D chemical structures of remdesivir (PubChem ID 121304016), abacavir (PubChem ID 441300), and sofosbuvir (PubChem ID 45375808) were used as standards in molecular docking studies.

### Target receptor preparation

RNA dependent RNA polymerase (RdRp) of five human pathogenic viral species were selected as target or receptor proteins in this study. Protein Data Bank (PDB) was used to find the molecular structures of RdRp of SARS-CoV-2 (PDB ID: 7B3B) [20], HIV-1 (6UK0) [23], Hepatitis C (4OOW) [24], Ebola (7YER) [25], and dengue virus (5K5M) [26] as receptor proteins. The receptor protein preparation includes adding hydrogen atoms, removing any extra ligand, solvent residues and water molecules, 3D protonation, energy minimized and then converted into pdbqt format through Macromolecule tool of PyRx. Protein preparation was carried out with AutoDock tool Vina [27] of PyRx allocated the rotatable bonds in the ligands. AutoGrid was utilized to create the grid parameters and dimensions. PROCHECK Ramachandran plots were used to assess the quality of protein structures.

### Molecular docking

Molecular docking was performed to screen strong antiviral secondary metabolites against RdRp of five different viruses using PyRx virtual screening software [27]. After prepration, the protein was imported into PyRx, converted to pdbqt format, and assigned as the receptor protein. A grid box was defined with AutoGrid dimensions of (X: 100.121, Y: 103.147, Z: 96.1872 for 7B3B) and with number of points (X: 73.67, Y: 76.55, Z: 75.77), (X: 38.81, Y: -26.22, Z: 28.81 for 6UK0) and with number of points (X: 63.12, Y: 98.28, Z: 60.89), (X: -25.68, Y: -7.11, Z: 27.45 for 4OOW) and with number of points (X: 59.01, Y: 59.29, Z: 76.31), (X:127.93, Y: 137.30, Z: 136.76 for 7YER) and with number of points (X: 91.58, Y: 88.81, Z: 97.15), and (X: -15.21, Y: -36.28, Z: -20.71 for 5K5M) and with number of points (X: 59.84, Y: 77.45, Z: 62.89) to increase the inquiry into the binding conformations of receptor proteins and ligands. The value of exhaustiveness was set to its default (i.e., 8). The docked poses with the lowest root mean square deviation (RMSD) and binding free energy were chosen. A significant evaluation criterion for screening prospective drugs against RdRp enzyme is the docking score between the receptor protein and ligand [28]. Lastly, the visualization of interactions between the receptor protein and important active compounds was done using the Biovia Discovery Studio 2021 [29] software.

### Drug scanning

The selected compounds were analyzed by an online tool available at (http://www.scfbio-iitd.res.in/software/drugdesign/lipinski.jsp/; accessed 15 April 2023) to check drug-likeness of these compounds with strong docking scores. Lipinski’s rule of five (Ro5) [30] identifies drug-like or non-drug-like compounds and predicts the success or failure of drug candidates in the preclinical stage based on five rules. According to Ro5, the molecular mass of the compound should be <500, hydrogen bond donors should be ≤5, hydrogen bond acceptors should be ≤10, log *P* value should be ≤5, and molecular refractive index should be between 40–130 [30].

### ADMET profiling

The online server admetSAR 2.0 [31] available at (http://lmmd.ecust.edu.cn/admetsar2/; accessed on 15 April 2023) was used to estimate the ADMET properties of selected compounds. ADMET based properties include absorption (plasma protein binding, human intestinal absorption, Caco-2 permeability), distribution (blood-brain barrier penetration, P-glycoprotein inhibitor, substrate), metabolism (CYP2D6 inhibitor, substrate, CYP2C9 inhibitor, substrate, CYP3A4 inhibitor, substrate, CYP1A2 inhibitor, CYP2C19 inhibitor, CYP inhibition promiscuity) and toxicity (carcinogenicity and Ames mutagenesis).

### Molecular dynamics simulation

One secondary metabolite against each receptor protein with best docking score and druggability properties was selected for molecular dynamics (MD) simulations. The MD simulation analysis of remdesivir (as a control) and RdRp of SARS-CoV-2 complex was also performed and the results were compared with those of best selected compounds. The MD simulation was run for 200 ns using Desmond (Schrödinger LLC) software for MD modeling in replicates [32]. First and last pose 2D images of MD trajectories were also obtained for a better comparison. The binding state of the ligand is predicted in static situations with the help of molecular docking and therefore only the earliest phase of the complex is used. The molecular docking is also important as it gives a static view of the bound ligand in the protein active site [33]. Newton’s classical equation of motion is integrated in MD simulation study which computes the movements of different atoms over time. In the current study, the MD simulation was run to predict the binding status of the ligand-protein complex in the physiological environment [34, 35].

The Maestro or Protein Preparation Wizard was used for the optimization and minimization of the ligand-protein complex. The System Builder tool was used to prepare all systems and a solvent model (i.e., transferable intermolecular interaction potential 3 points (TIP3P)) was selected with an orthorhombic box [36, 37]. In the MD simulation study, the OPLS 2005 force field was employed and the counter ions were introduced for neutralizing the models [38]. NaCl (0.15 M) was added for representing the physiological conditions [36]. The NPT ensemble with 310 K of temperature and 1 atm of pressure was adjusted for the time of simulation. The models were relaxed before starting simulation and the trajectories were saved after every 100 ps for examination. The protein RMSD values were compared with those of ligands over simulation time to verify the stability of MD simulation.

## Results

### Screening through molecular docking

**Fig 1:**
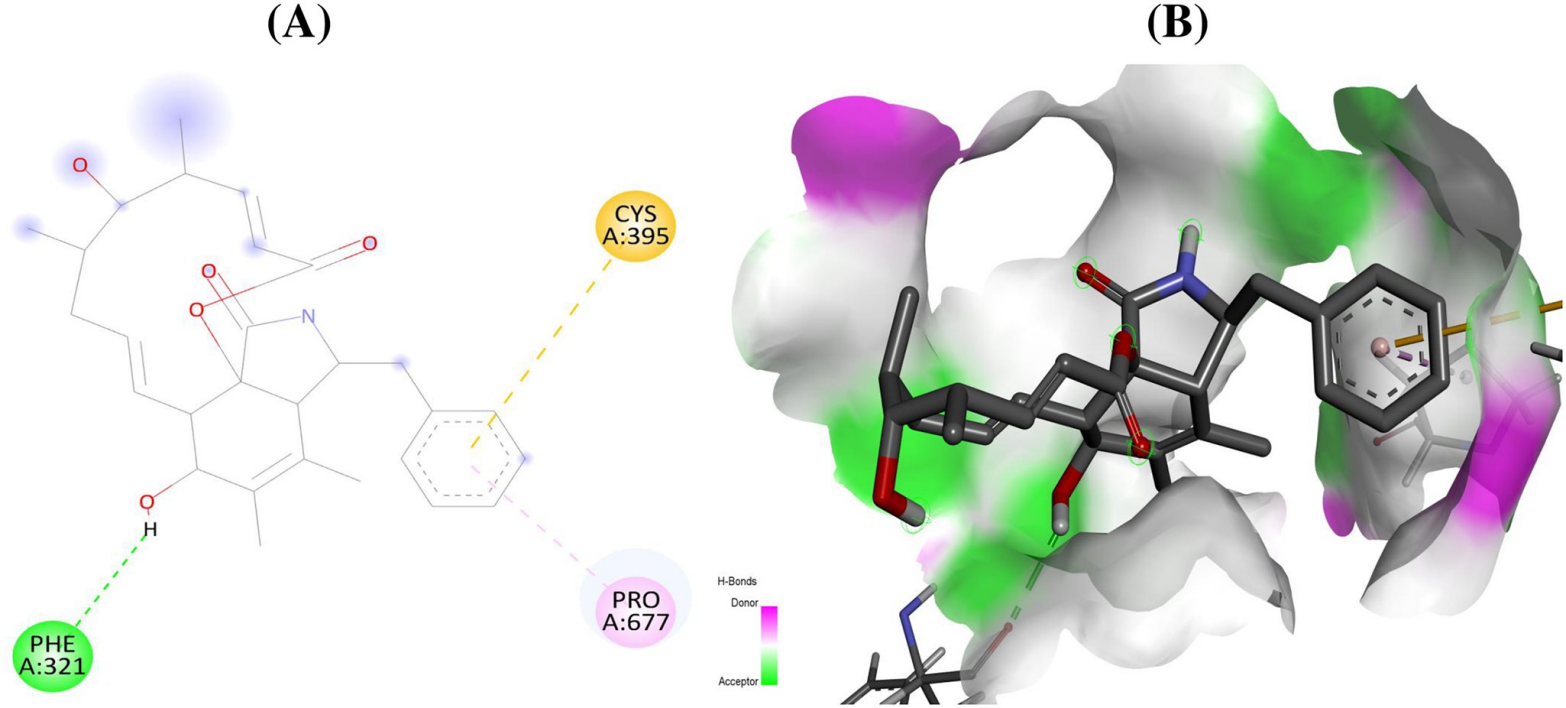
Interactions (A) and binding pattern (B) of cytochalasin Z8 (11518356) with RdRp of SARS-CoV-2 (7B3B) as a receptor.

**Fig 2:**
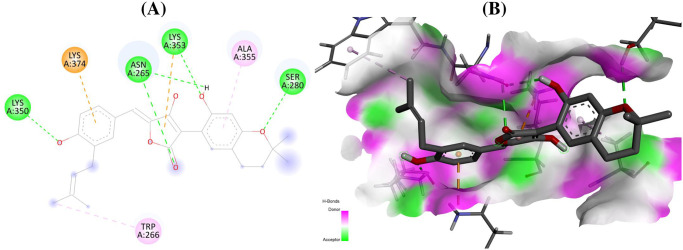
Interactions (A) and binding pattern (B) of aspulvinone D (54678424) with RdRp of HIV-1 (6UK0) as a receptor.

For the initial virtual screening, 200 secondary metabolites were docked to RdRp of five selected target proteins. By integrating with active site residues, the protein-ligand docking binding pattern and docking scores filtered out the top five hits as strong active drug candidates against RdRp enzyme. All of the best-chosen secondary metabolites in this investigation demonstrated potent interactions with the active site residues of the catalytic sites of the RdRp enzyme (Table 1). The secondary metabolites cytochalasin Z8 had lowest docking score of –8.9 (kcal/mol) against RdRp of SARS-CoV-2 (7B3B) comparable to standard (remdesivir: –7.4 kcal/mol) interacted with the binding pocket residues (PheA:321, CysA:395, ProA:677) (Fig 1), aspulvinone D showed docking score of –9.2 (kcal/mol) against RdRp of HIV-1 (6UK0) while shared common interacting binding pocket residues (LysA:350 and LysA:374) with standard (remdesivir: –7.7 kcal/mol) in the current investigation (Fig 2).

**Table 1:** Computation binding energy profiling of secondary metabolites as potential drug candidates against RdRp of five different viruses.

RdRp with PDB ID	Ligand	PubChem ID	Docking score (kcal/mol)	Interacting residues
SARS-CoV-2 (7B3B)	Cytochalasin Z8	11518356	–8.9	PheA:321, CysA:395, ProA:677
	Remdesivir (Control)	121304016	–7.4	LysA:780, AlaA:706, ValA:776, SerA:709, ThrA:710, TyrA:32, LysA:47
HIV-1 (6UK0)	Aspulvinone D	54678424	–9.2	LysA:350, LysA:374, AsnA:265, LysA:353, AlaA:355, SerA:280, TrpA:266
	Remdesivir (Control)	121304016	–7.7	LysA:350, GlyA:352, GlnA:269, LysA:374, IleA:94
Hepatitis C (4OOW)	Talaromyolide D	146683236	–9.9	GlyA:557, AspA:559, ProA:93, GlyA:449
	Remdesivir (Control)	121304016	–8.4	CysA:451, AspA:409, ProA:93, GlyA:557, LysA:141, ValA:144, TrpA:397, IleA:160, AlaA:97
Ebola (7YER)	Aspulvinone D	54678424	–9.9	AsnA:558, ArgA:561, LeuA:638, AspA:742
	Remdesivir (Control)	121304016	–8.6	ArgA:561, GluA:299, TyrA:800, LysA:803, LysA;296, TheA:297, ProA:300
Dengue (5K5M)	Talaromyolide D	146683236	–9.2	LysA:283, ValA:452, MetA:477, TrpA:478, ArgA:579
	Remdesivir (Control)	121304016	–7.5	LysA:402, AsnA:406, ArgA:482, GluA:485, GluA:494

**Fig 3:**
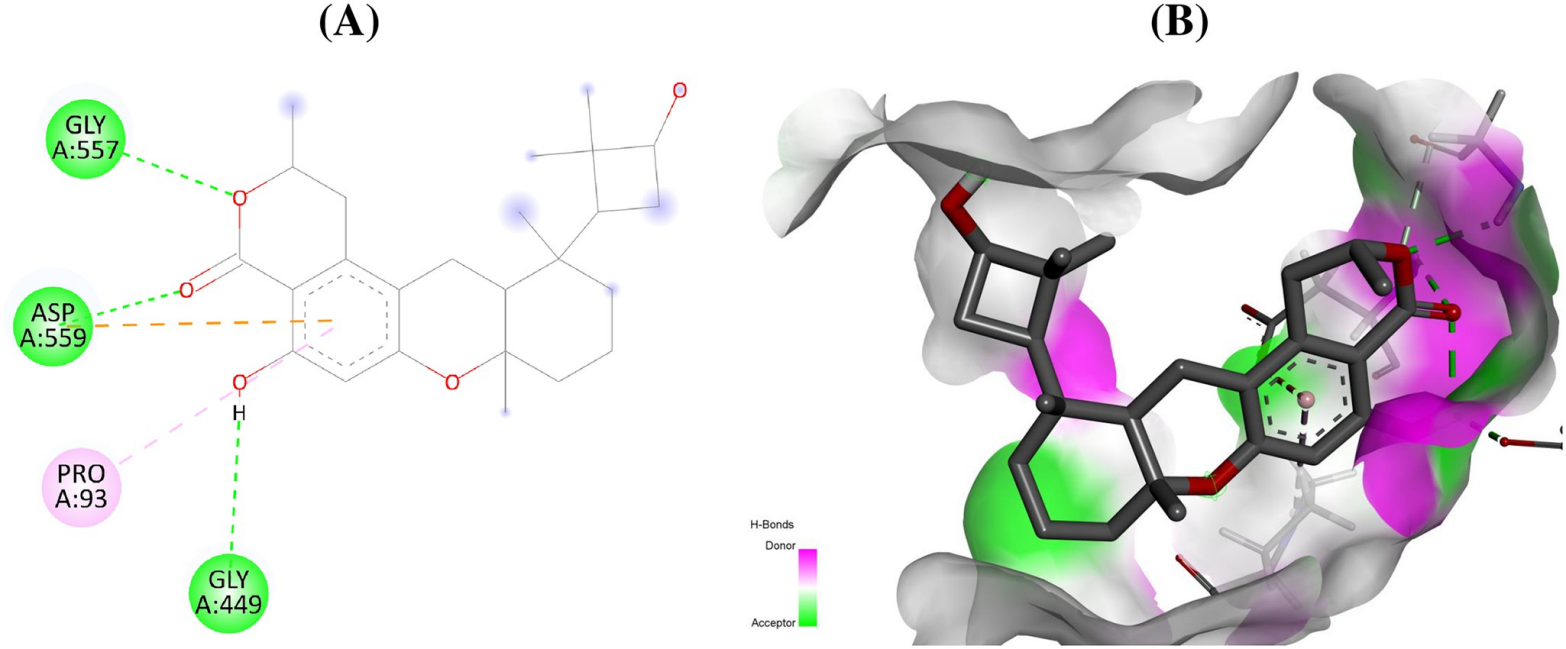
Interactions (A) and binding pattern (B) of talaromyolide D (146683236) with RdRp of hepatitis C (4OOW) as a receptor.

**Fig 4:**
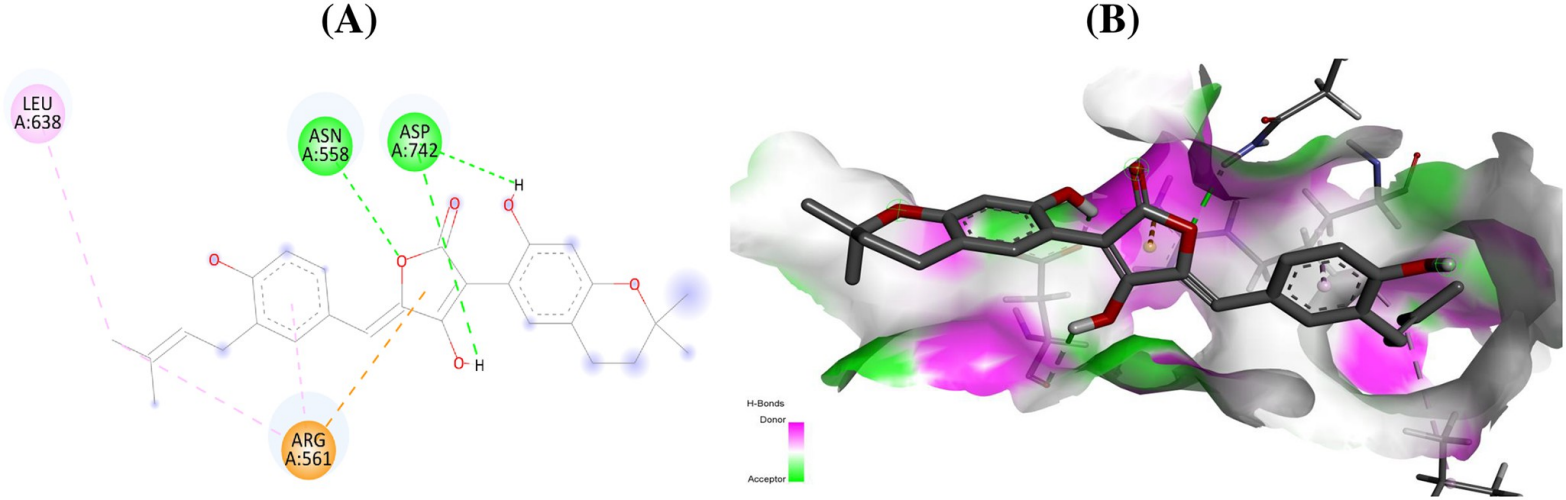
Interactions (A) and binding pattern (B) of aspulvinone D (54678424) with RdRp of Ebola (7YER) as a receptor.

**Fig 5:**
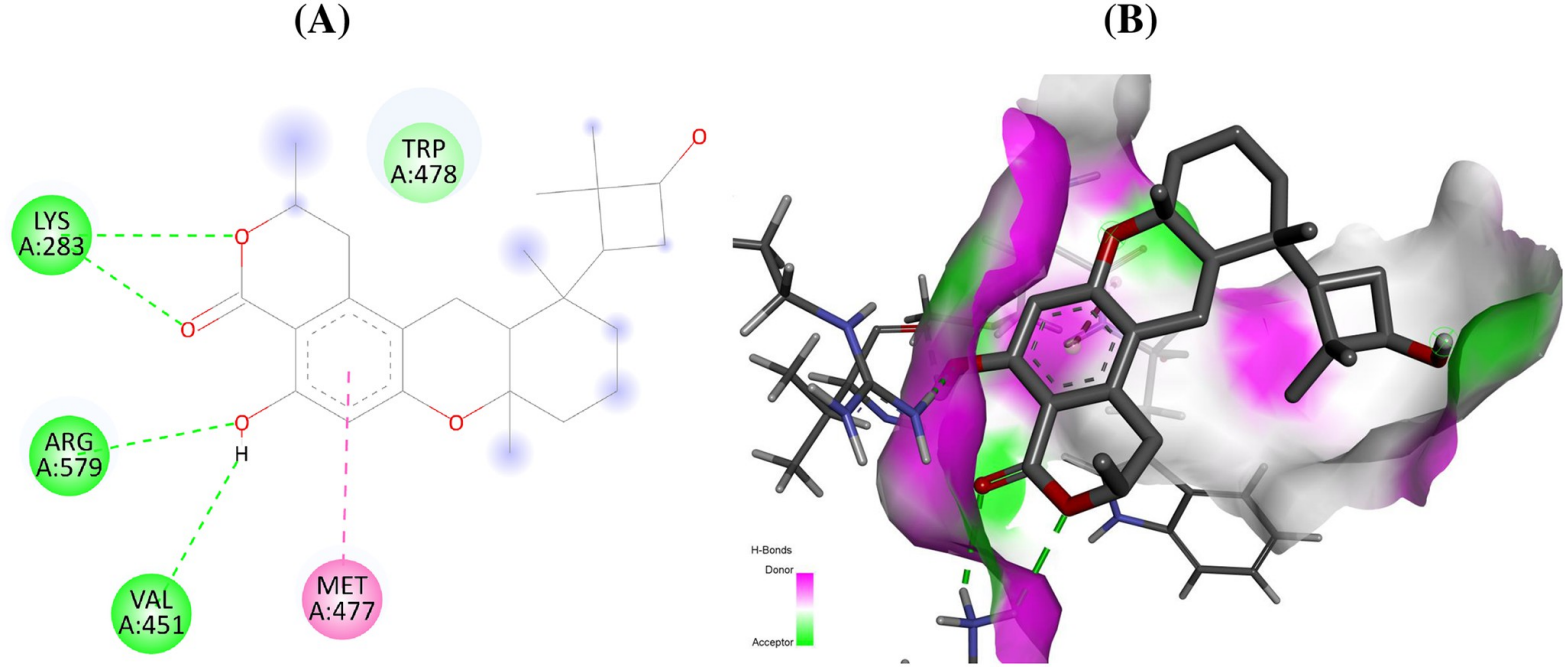
Interactions (A) and binding pattern (B) of talaromyolide D (146683236) with RdRp of dengue (5K5M) as a receptor.

**Fig 6:**
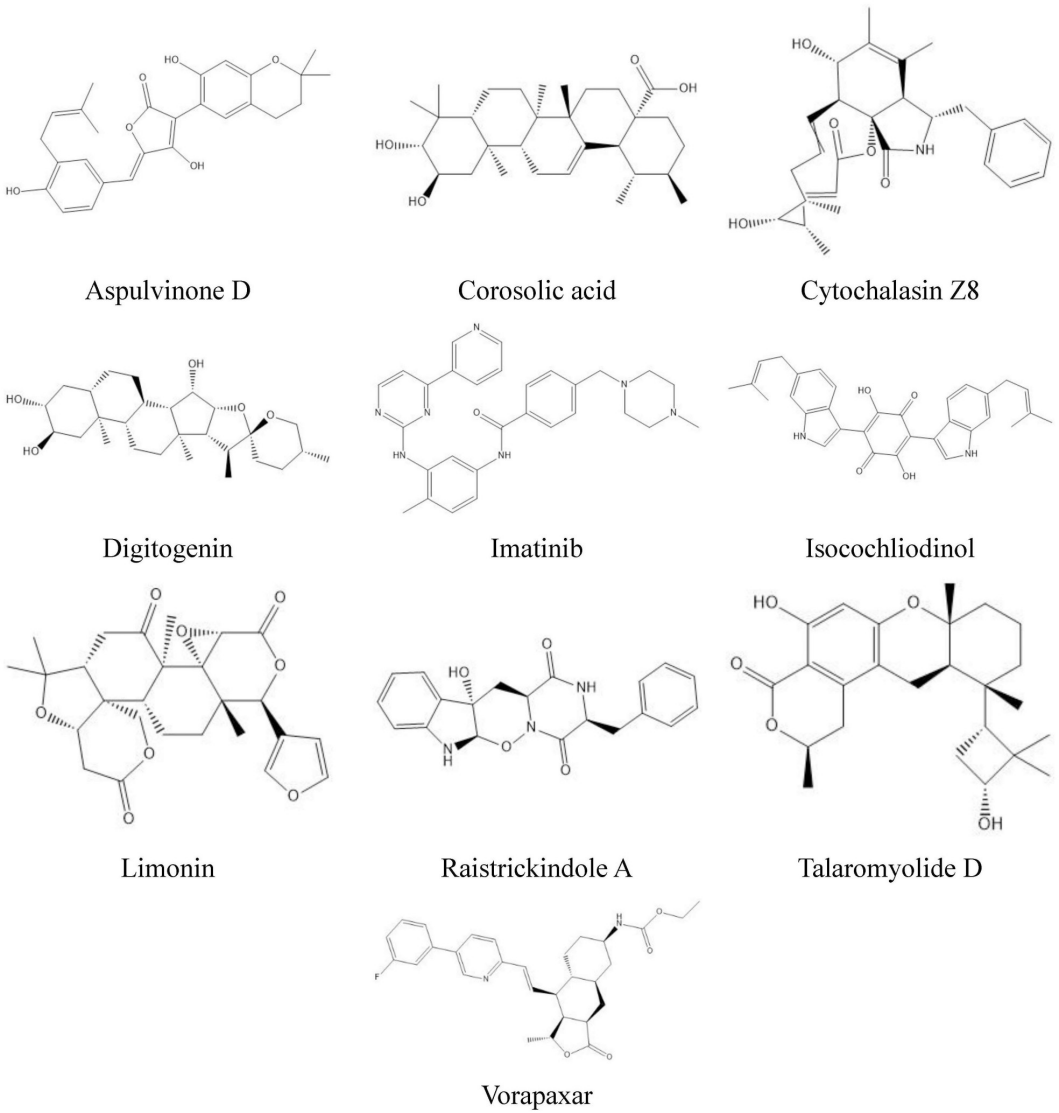
Chemical structures of the best active antiviral compounds found via molecular docking study.

The compound talaromyolide D established conventional hydrogen bonds with GlyA:557, AspA:559, and GlyA:449 residues of RdRp enzyme of hepatitis C with a docking score of –9.9 kcal/mol (Table 1, Fig 3). Similarly, for RdRp of Ebola virus, the amino acid residues AsnA:558, AspA:742 form conventional hydrogen bond with aspulvinone D with a docking score of –9.9 kcal/mol (Fig 4). The secondary metabolite talaromyolide D also maintained the lowest possible S-score of –9.2 kcal/mol against RdRp enzyme of dengue virus while established conventional hydrogen bonds with amino acids LysA:283, ValA:452, TrpA:478, ArgA:579 (Fig 5). The chemical structures of the best active antiviral compounds against five selected receptor proteins are given in Fig 6. The quality of 3D structures of receptor proteins was checked through PROCHECK Ramachandran plots and is given in S11 Fig in S1 File.

The other selected natural biomolecules showed binding affinity ranging from –9.4 to –9.1 kcal/mol against RdRp enzyme of SARS-CoV-2 (S1 Table, S1 Fig in S1 File), –9.4 to –8.9 kcal/mol against RdRp enzyme of HIV-1 (S1 Table, S2 Fig in S1 File), –10.2 to –9.7 kcal/mol against RdRp enzyme of hepatitis C (Table 1, S3 Fig in S1 File), –10.7 to –9.7 kcal/mol against RdRp enzyme of Ebola (S1 Table, S4 Fig in S1 File), and –9.8 to –9.2 kcal/mol against RdRp enzyme of dengue virus (S1 Table, S5 Fig in S1). The binding affinity of screened natural metabolites was much lower than all three selected standard chemical drugs approved for the viral treatment by targeting RNA dependent RNA polymerase enzyme (S2 Table, S6-S10 Figs in S1 File).

### Drug-likeness and ADMET properties

All the selected secondary metabolites followed the drug-likeness properties (Ro5) with no violations except corosolic acid, imatinib, isocochliodinol, and vorapaxar (Table 2). The ADMET based attributes of drug candidates for the biological availability, metabolism, and toxicity were predicted using admetSAR tool in order to further evaluate the pharmacological potential of these secondary metabolites. For a possible medication, a candidate compound that passes the ADMET assessment can be regarded as safe. The findings showed that the selected compounds were non-carcinogens. Overall, the chosen ligands were determined to be suitable as potential medication candidates (Table 3).

**Table 2:** Evaluation of drug-like properties by rule of five of selected secondary metabolites against viral RdRp enzyme.

Compound	ID	Classification	MW	HBD	HBA	LogP	MR	Violation
Aspulvinone D	54678424	Transferase Enzyme	448	3	6	5.57	125.9	0
Corosolic acid	6918774	Terpenes	472	3	4	6.06	134.0	2
Cytochalasin Z8	11518356	Cytochalasin	465	3	6	3.10	129.4	0
Digitogenin	441886	Sterol	448	3	5	3.73	120.5	0
Imatinib	5291	Carboxylic Acid	493	2	8	4.40	146.2	1
Isocochliodinol	474301	Indole	506	4	4	6.87	150.8	3
Limonin	179651	Limonoid	470	0	8	3.13	114.1	0
Raistrickindole A	145720909	Peptide alkaloid	365	3	7	0.89	96.1	0
Talaromyolide D	146683236	Meroterpenoids	414	2	5	4.40	112.7	0
Vorapaxar	10077130	Lactone	492	1	6	5.62	134.0	2

MW: Molecular weight; HBD: Number of hydrogen bond donors; HBA: Number of hydrogen bond acceptors; log P: the logarithm of octanol/water partition coefficient; MR: Molar refractivity.

**Table 3:** ADMET related drug-like parameters of the best selected secondary metabolites against viral RdRp enzyme.

Parameters	Aspulvinone D	Corosolic acid	Cytochalasin	Digitogenin	Imatinib	Isocochliodinol	Limonin	Raistrickindole A	Talaromyolide D	Vorapaxar
PubChem ID	54678424	6918774	11518356	441886	5291	474301	179651	145720909	146683236	10077130
**Absorption**										
Caco-2 Permeability	-	-	-	-	-	-	-	-	+	-
HIA	+	+	+	+	+	+	+	+	+	+
HOB	-	+	-	-	+	+	+	-	-	+
Pgp inhibitor	Inh	NI	NI	NI	Inh	Inh	Inh	NI	NI	Inh
Pgp substrate	NS	NS	Sub	NS	Sub	NS	NS	NS	NS	Sub
**Distribution**										
BBB Penetration	-	-	-	-	+	+	+	-	-	+
PPB	0.93	0.79	0.96	0.69	1.01	1.02	0.81	1.03	0.67	0.87
**Metabolism**										
CYP1A2 inhibitor	NI	NI	IN	NI	IN	Inh	IN	IN	NI	Inh
CYP2C19 inhibitor	NI	NI	IN	NI	NI	Inh	NI	IN	NI	Inh
CYP2C9 inhibitor	Inh	NI	IN	NI	NI	Inh	NI	IN	NI	Inh
CYP2C9 substrate	NS	NS	NS	NS	NS	Sub	NS	NS	Sub	NS
CYP2D6 inhibitor	NI	NI	IN	NI	NI	Inh	NI	NI	NI	NI
CYP2D6 substrate	NS	NS	NS	NS	NS	NS	NS	NS	NS	NS
CYP3A4 inhibitor	IN	NI	IN	NI	NI	NI	Inh	IN	NI	Inh
CYP3A4 substrate	Sub	Sub	Sub	Sub	Sub	Sub	Sub	Sub	Sub	Sub
**Toxicity**										
AMES Toxicity	-	-	-	-	-	-	-	-	-	-
Carcinogenicity	-	-	-	-	-	-	-	-	-	-

BBB: blood-brain barrier; PPB: plasma protein binding; HIA: human intestinal absorption; HOB: human oral bioavailability; Pgp substrate: P-glycoprotein substrate; Pgp Inhibitor: P-glycoprotein inhibitor; NS: no substrate; NI: noninhibitor; Sub: substrate; Inh: inhibitor; -: negative; +: positive.

### Molecular dynamics simulation analysis

**Fig 7:**
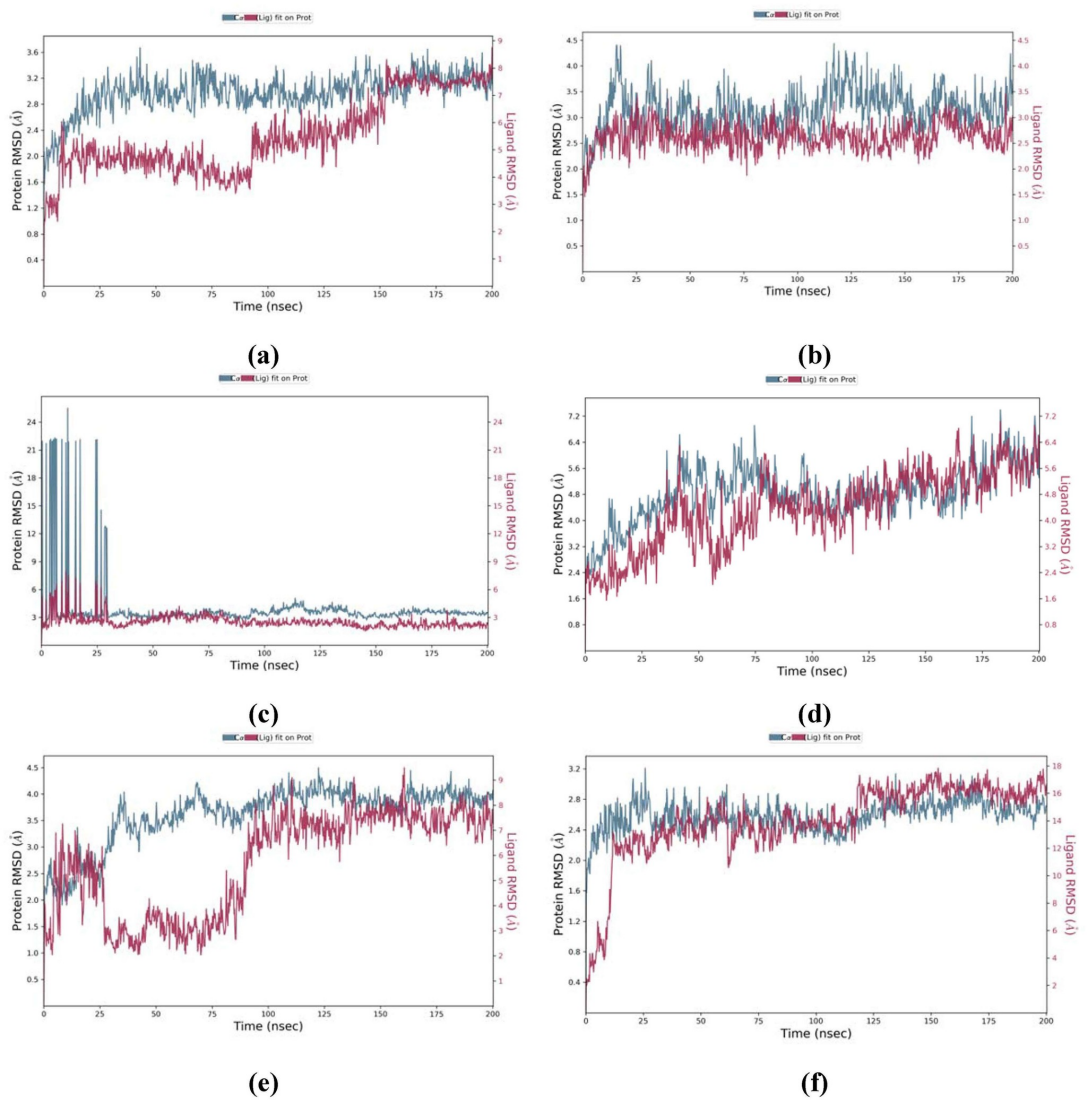
Root-mean-square deviation (RMSD) values of selected receptor proteins with their best ligands. (a) RMSD of the C-alpha atoms of RdRp of SARS-CoV-2 and cytochalasin Z8; (b) RMSD of the C-alpha atoms of RdRp of HIV-1 and aspulvinone D with time; (c) RMSD of the C-alpha atoms of RdRp of hepatitis C with talaromyolide D; (d) RMSD of the C-alpha atoms of RdRp of Ebola with aspulvinone D; (e) RMSD of the C-alpha atoms of RdRp of dengue with talaromyolide D; (f) RMSD of C-alpha atoms of RdRp of SARS-CoV2 and remdesivir as a control. The variation of protein RMSD is shown on the left Y-axis through time. The variation of ligand RMSD is shown on the right Y-axis through time.

Molecular dynamics (MD) simulation analysis was performed to verify the stability of the best ligand against each receptor protein on the basis of findings of molecular docking and druggability analysis. All ligands were found to be firmly bound within the binding pockets of their respective receptor proteins (Fig 7). The ligand-protein complexes did not show drastic fluctuations in their RMSD values throughout simulation period of 200 ns. In ligand-protein complex of RdRp of SARS-CoV-2 and cytochalasin Z8, the protein was found to be stable throughout simulation time while the ligand fluctuated a little after 90 ns from 4Å to 8Å which is quite acceptable. The RdRp of HIV-1 and aspulvinone D complex was revealed to be sufficiently stable throughout the simulation time of 200 ns. The ligand-protein complex of RdRp of hepatitis C and talaromyolide D showed fluctuations in the RMSD values of both protein and ligand till 30 ns but later both gained stability for the rest of the simulation time. Both protein and the ligand in RdRp of Ebola with aspulvinone D complex were overlapped throughout simulation time and the RMSD values showed that the complex was stable throughout simulation time.

In ligand-protein complex of talaromyolide D and RdRp of dengue protein, the RMSD value for Cα-atoms of protein was in the range of 2 to 4Å with a change of 2Å which is acceptable, however, the ligand talaromyolide D showed fluctuations in RMSD values till 82 ns and then the ligand attained stability for the rest of the simulation time. As a control, an antiviral drug remdesivir was also used in the MD simulation study complexed with RdRp of SARS-CoV2. The complex attained stability after 10 ns and the ligand was found to be firmly attached to the receptor protein for the rest of the simulation time. All selected ligand-protein complexes showed stable binding through simulation time and the ligands were firmly bound within the original pockets of their respective target proteins.

**Fig 8:**
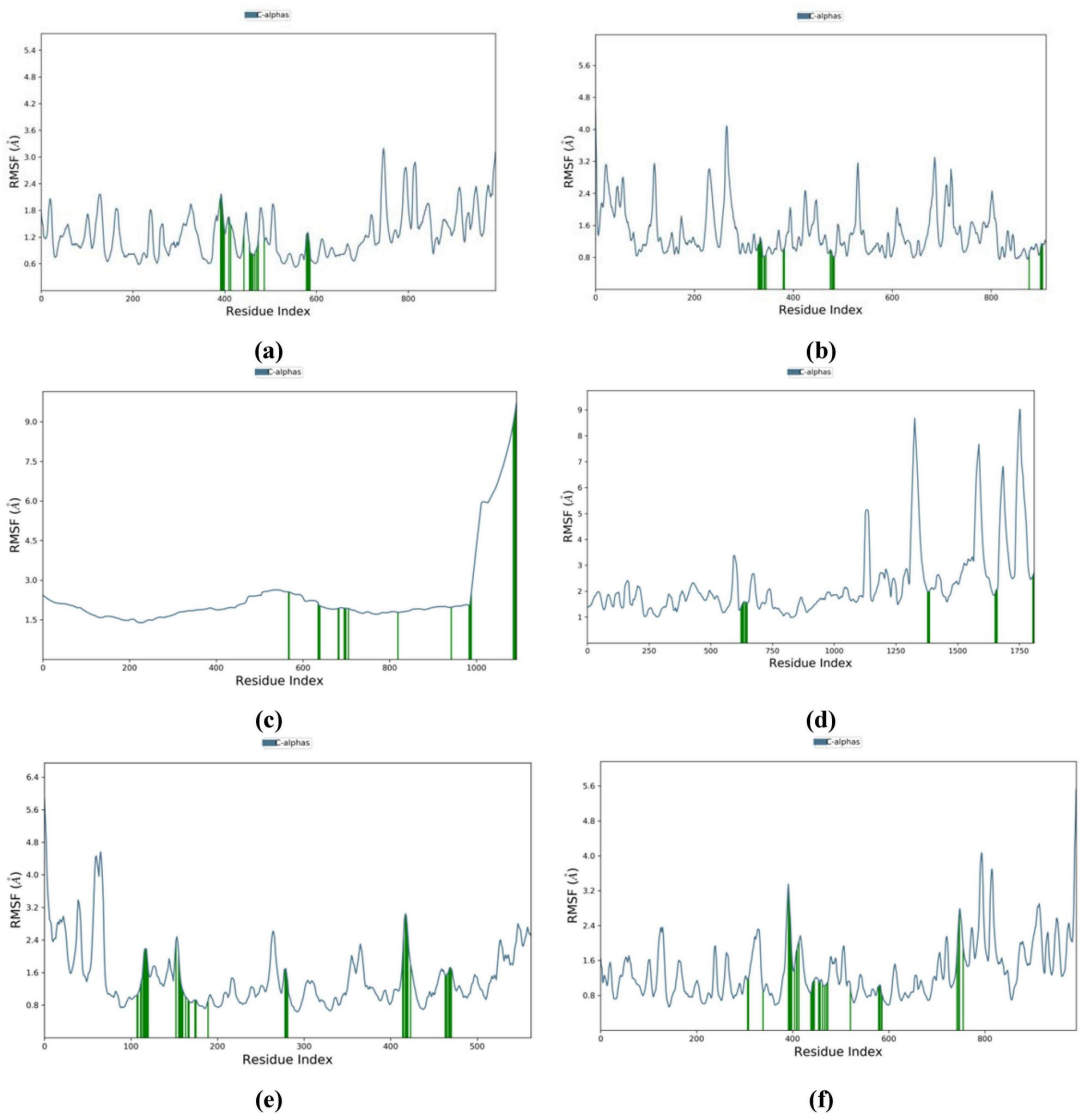
Residue wise root mean square fluctuation (RMSF) of RdRp of selected receptor proteins. (a) SARS-CoV-2 and cytochalasin Z8 complex, (b) HIV-1 and aspulvinone D complex, (c) hepatitis C and talaromyolide D complex, (d) Ebola and aspulvinone D complex, (e) dengue and talaromyolide D complex, (f) SARS-CoV2 and remdesivir (control) complex.

The analysis of root mean square fluctuation (RMSF) of receptor proteins complexed with their best selected ligands showed stable peaks throughout simulation time of 200 ns (Fig 8). The findings showed that the ligand-protein complexes are stable.

**Fig 9:**
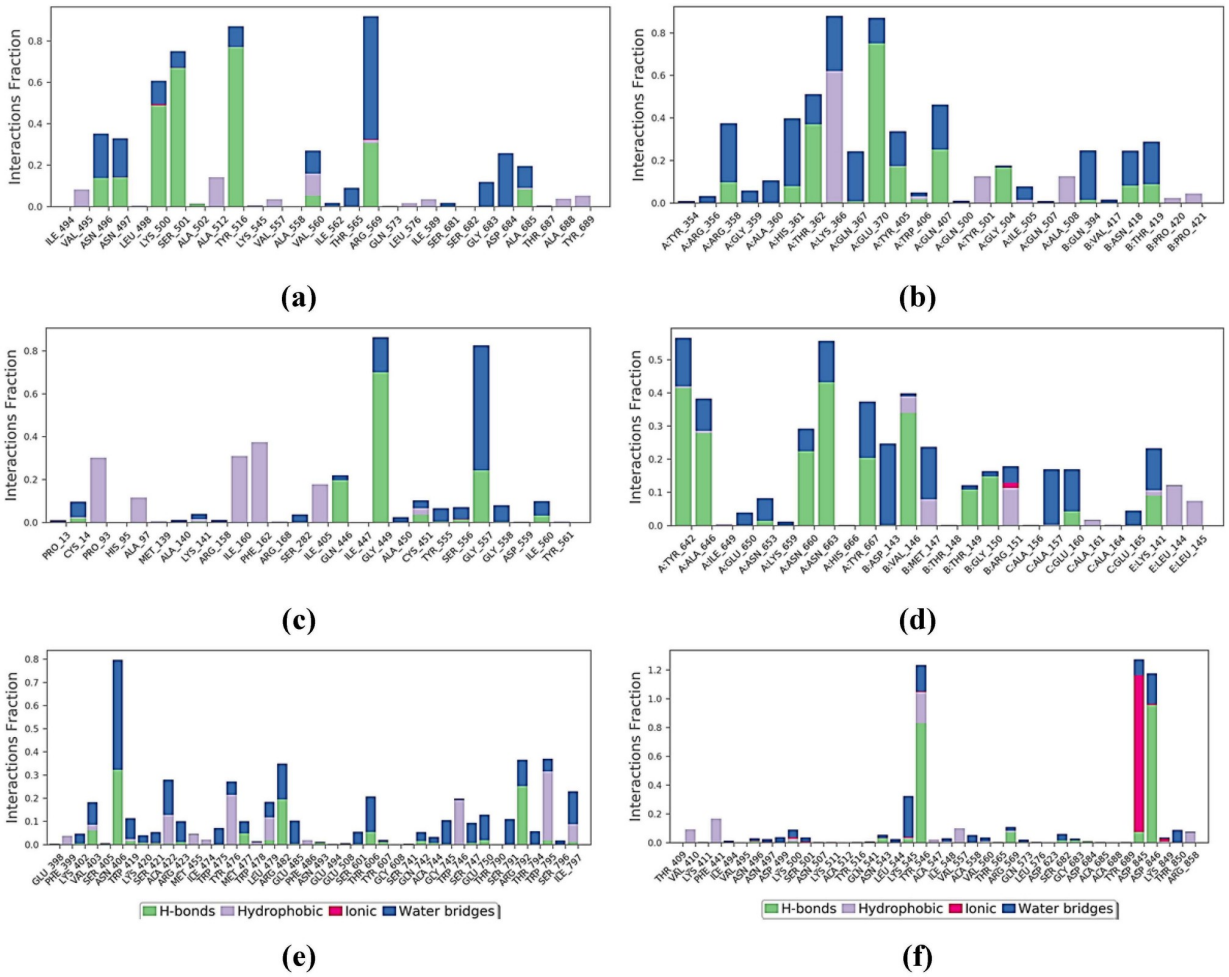
Protein-ligand contact histograms (H-bond, hydrophobic, ionic, and water bridges) of; (a) RdRp of SARS-CoV-2 and cytochalasin Z8 complex, (b) RdRp of HIV-1 and aspulvinone D complex, (c) RdRp of hepatitis C and talaromyolide D complex, (d) RdRp of Ebola and aspulvinone D complex, (e) RdRp of dengue and talaromyolide D complex, (f) RdRp of SARS-CoV2 and remdesivir (control) complex.

The PLIF (protein ligand interaction fingerprints) of RdRp of SARS-CoV-2 and cytochalasin Z8 complex before and after simulation revealed that amino acids Asn496, Asn497, Lys500, Ser501, Tyr516, Arg569, and Ala685 are involved in making hydrogen bonds with the ligand and are therefore important to stabilize the complex (Fig 9a). In RdRp of HIV-1 and aspulvinone D complex, the amino acids Arg358, His361, Thr362, Glu370, Tyr405, Gln407, Gly504, Asn418, and Thr419 were found to be involved in stabilizing the complex through H-bond formation (Fig 9b). Similarly, in case of RdRp of hepatitis C and talaromyolide D complex, the amino acids Ile405, Gly449, and Gly557 were involved in making H-bonds with the ligand molecule (Fig 9c). The amino acids Tyr642, Ala646, Asn660, Asn663, Tyr667, of chain A and amino acids Val146, Thr149, Gly150, Glu160, and Lys141 of chain B of RdRp of Ebola virus were involved in making H-bonds with ligand aspulvinone D to stabilize the complex (Fig 9d). Similarly, the amino acids Val403, Asn406, Met477, Arg482, Thr606, and Arg792 stabilized the RdRp of dengue-talaromyolide D complex through hydrogen bonding (Fig 9e). In case of control, the amino acids Tyr546, Arg569, Asp845, and Asp846 were involved in stabilizing the ligand-protein complex of RdRp of SARS-CoV2 and remdesivir (Fig 9f).

**Fig 10:**
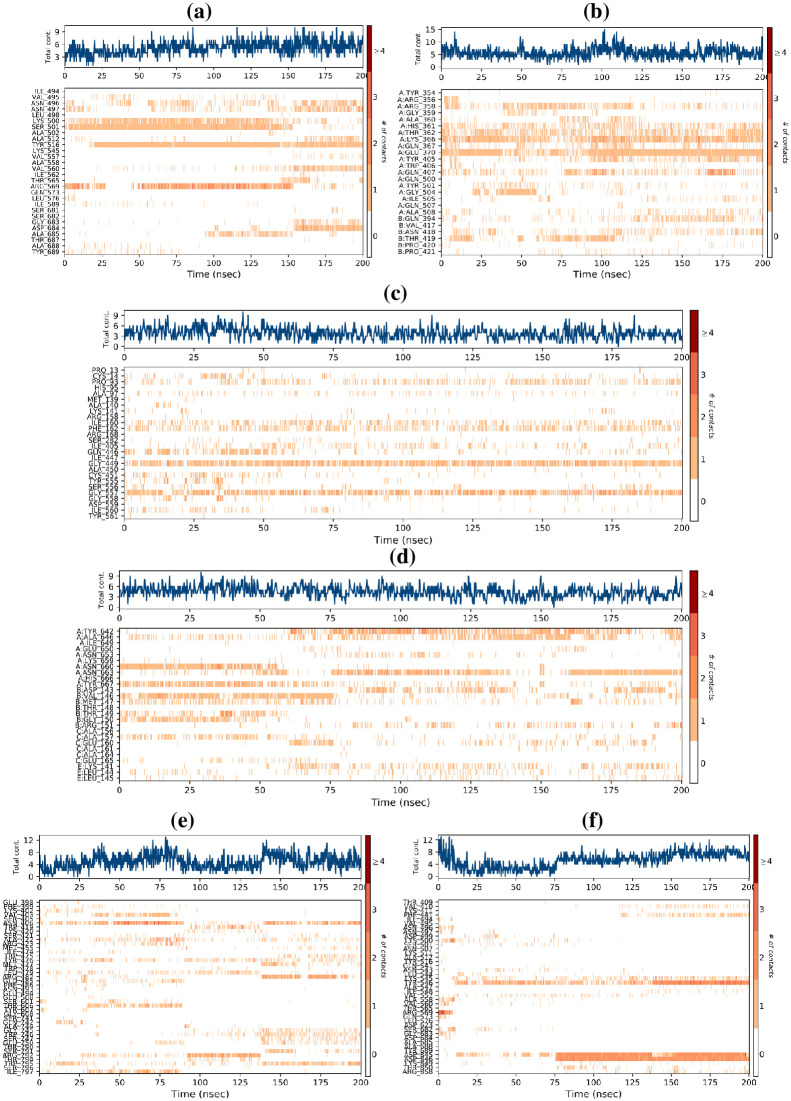
A timeline representation of the interactions and contacts (H-bonds, hydrophobic, ionic, and water bridges) between; (a) RdRp of SARS-CoV-2 and cytochalasin Z8 complex, (b) RdRp of HIV-1 and aspulvinone D complex, (c) RdRp of hepatitis C and talaromyolide D complex, (d) RdRp of Ebola and aspulvinone D complex, (e) RdRp of dengue and talaromyolide D complex, (f) RdRp of SARS-CoV-2 and remdesivir complex as a control.

The complexes of the best selected ligand molecules with five RdRp proteins were also found to be stable through simulation time of 200 ns through the analysis of hydrogen bond interaction stability (Fig 10). The analysis validates the selected ligands to be used as potential candidates against five selected viral proteins.

**Fig 11:**
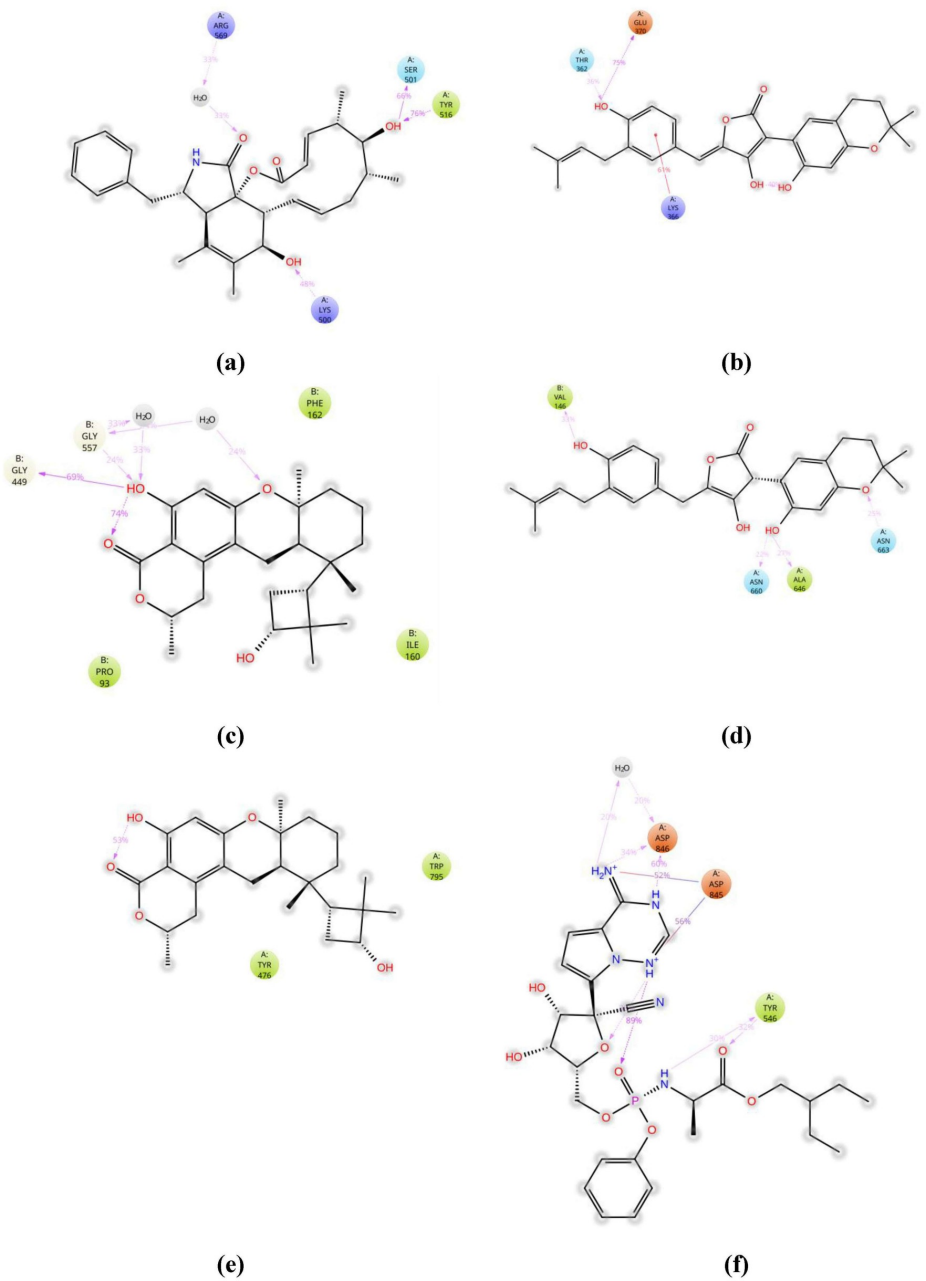
Ligand atom interactions with protein residues. (a) interaction of cytochalasin Z8 atoms with RdRp of SARS-CoV-2 protein residues; (b) interaction of aspulvinone D atoms with RdRp of HIV-1 protein residues; (c) interaction of talaromyolide D atoms with RdRp of hepatitis C protein residues; (d) interaction of aspulvinone D atoms with RdRp of Ebola protein residues; (e) interaction of talaromyolide D atoms with RdRp of dengue protein residues; (f) interaction of remdesivir atoms with RdRp of SARS-CoV-2 protein residues.

For the course of trajectory, the stacked bar charts were standardized. According to these stacked bar charts, the contact value of 1.0 shows exact interaction retained for 100% of simulation time. The interactions of best selected ligand molecules with their receptor proteins are shown in Fig 11. Only those interactions which lasted >30% of simulation time in their respective trajectories are given. First and last pose 2D images of MD tranjectoies of the best selected ligand molecules were also obtained and comprated to those of remdesivir as a control (S12, S13 Figs in S1 File).

**Fig 12:**
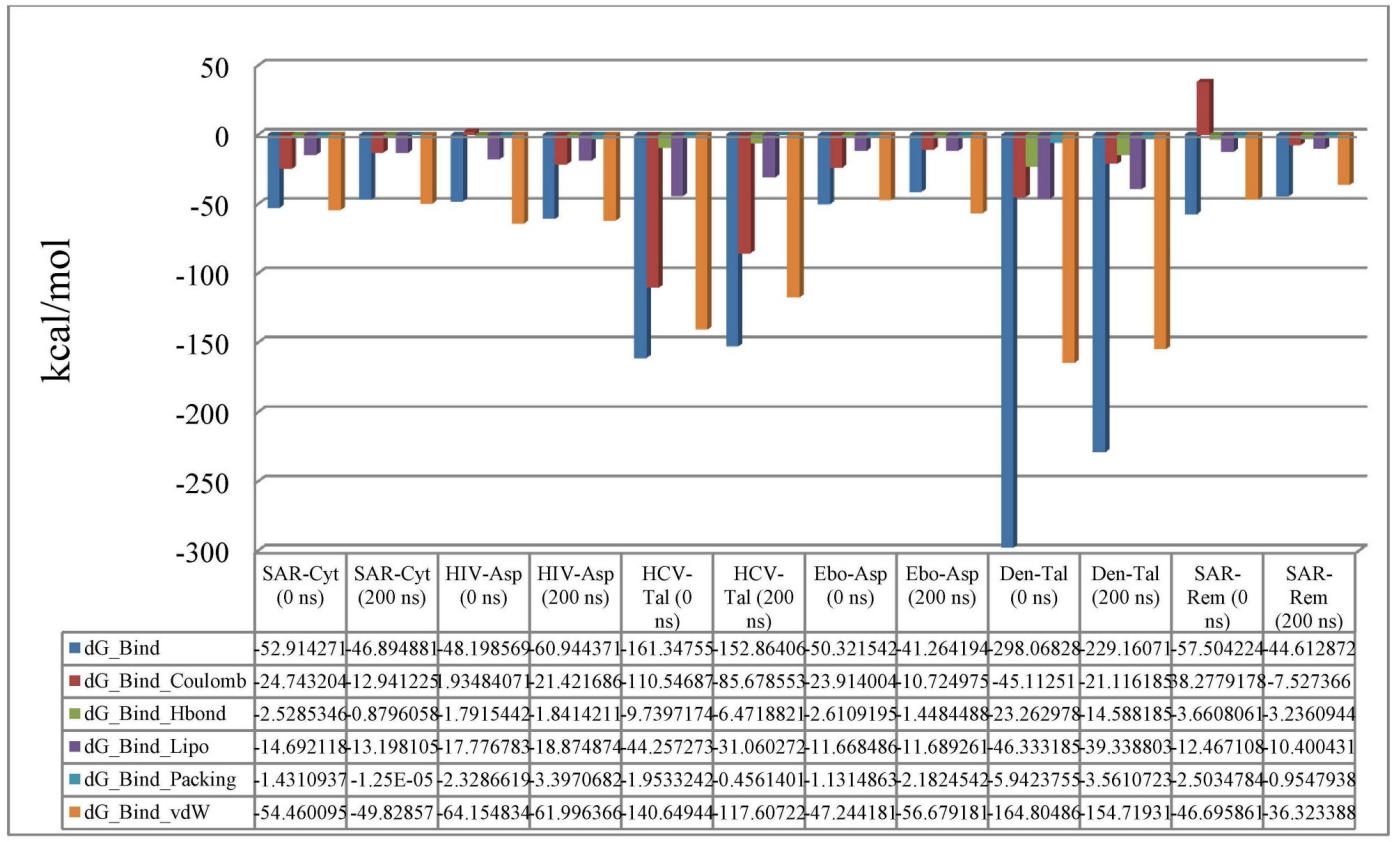
MM-GBSA method was employed for the prediction of binding energy between five receptor proteins and their respective best ligands. SAR: SARS-CoV-2, Cyt: Cytochalasin Z8, HIV: HIV-1, Asp: Aspulvinone D, HCV: Hepatitis C, Tal: Talaromyolide D, Ebo: Ebola, Den: Dengue, Rem: Remdesivir.

The MM-GBSA or molecular mechanics generalized born surface area was also determined for MD simulation (Fig 12). For cytochalasin Z8 and RdRp of SARS-CoV-2 complex, the average ΔG was –49.39 kcal/mol, the ΔG range was –52.91 to –46.89, and the value of standard deviation was 5.42. The average ΔG for aspulvinone D complex with RdRp of HIV-1 protein was found to be –62.50 with its range from –48.19 to –60.94 and standard deviation of 10.67. The complex of talaromyolide D with RdRp of hepatitis C showed average ΔG value of –147.10 with range from –161.34 to –152.86 and standard deviation of 25.18. The average ΔG value for aspulvinone D complexed with RdRp of Ebola virus was found to be –52.73 with range form –50.32 to –41.26 and standard deviation of 7.75. Similarly, for talaromyolide D and RdRp of dengue virus complex, the average ΔG value was –257.41 and the standard deviation value was 17.44 and the range of ΔG was found to be from –298.06 to –229.16. As a control, remdesivir and RdRp of SARS-CoV-2 complex, the average ΔG was –47.77 kcal/mol, the ΔG range was –57.50 to –44.61, and the value of standard deviation was 7.65. The majority of lower binding energy values of best selected ligands with their receptor proteins are validating that the residues of all receptor proteins contributed significantly in stabilizing the ligand molecules near their binding sites.

## Discussion

Prior to performing laboratory trials, researchers can analyze the binding mechanisms of different ligands with the aid of novel computational techniques. Modern drug discovery methods such as docking of molecules and dynamic simulation provide essential information on the stability and active site targeting of possible medicinal products against certain receptor proteins [17]. These bioinformatics tools help to identify the target molecules and their validation as well as enhance the accuracy and efficiency of treatment to cure these fatal viral diseases in very short time. The secondary metabolites such as alkaloids, phenolics, terpenoids and other bioactive compounds have proven their antiviral activity due to their vast structural efficacy [39]. The molecular docking method has been used to demonstrate the ability of several candidates for ligand molecules to bind to various receptor proteins. Various docking strategies have been used depending on the type and degree of analysis as well as the receptor-ligand type. The interactions, energy verifications, and patterns of ligand binding to the receptor moieties’ catalytic site are carried out by the docking studies. The testing of various collections of ligand compounds towards various targets, such as proteins, can be aided by computer-assisted drug discovery [40]. Maximum binding pocket occupancy, powerful H-bonds, and a minimal energy structure support the possibility of ligand molecules interacting with receptor protein active residues. Before beginning experimental research, computational studies assist scientists in estimating the outcomes of the prospective study.

Diverse geographic areas and the human population have employed wild flora for therapeutic purposes for many years. Natural secondary metabolites used to maintain health and treat a variety of disorders have been found to include a wide spectrum of chemical substances with bioactive components [2]. Secondary metabolites with a variety of biological roles are produced by plants and microorganisms. These secondary metabolites fall into categories based on numerous characteristics, including chemical structure (the existence of rings or sugars), composition (the presence or absence of nitrogen), solubility in organic solvents or water, and metabolic process. Carotenoids, phytosterols, phenolics, alkaloids, nitrogen compounds, and organosulfur compounds are among them. Each class is subsequently subdivided into subclasses. Phenolics, in particular, are a diverse category of chemicals, with the phenolic hydroxyl groups serving as the common structural component. These are commonly found conjugated with sugars and organic acids and are classified into seven major groups (i.e., phenolic acids, hydrolysable tannins, flavonoids, stilbenes, coumarins, curcuminoids, and lignans) [9, 41].

According to recent research, some of these natural substances can lower the risk of numerous chronic illnesses caused by retroviruses, because of their antibacterial, antioxidant, and anti-inflammatory and immunomodulatory properties [42]. Recent breakthroughs in analytical methodologies, such as synthetic biology, metabolic engineering, and metabolomics, as well as computer tools and databases, are providing significant tools for natural chemical drug discovery. Natural products have been shown to have significant antiviral activity, typically against many viral families, implying that they could be beneficial as broad-spectrum antiviral medicines [43]. A drug’s antiviral activity can influence viral DNA and RNA synthesis, viral reproduction or virus entry and is influenced by viral structure and the replication cycle. The antiviral activity of secondary metabolites can be tested using several biological assays to determine cytotoxicity, cytopathic effect suppression, and the ability to stop viral spread from cell to cell, hence restricting and/or combating viral circulation.

Some natural elements present in plant-based foods and dietary supplements show effectiveness against SARS-CoV-2 via affecting cytokine production, modulating cell signaling pathways relevant to inflammation, and even directly interacting with virus targets [44]. This has been established through the use of *in vitro* and *in silico* molecular docking studies to determine binding strength. Furthermore, the aflavin from *Camellia sinensis* (tea) has pharmacological activity against SARS-CoV-2 via binding to RNA-dependent RNA polymerase [45, 46]. In this study, aspulvinone D showed comparable significant interaction with RdRp of SARS-CoV-2. Apigenin has been shown to inhibit SARS-CoV 3CLpro, whereas luteolin adheres to the exterior of the SARS-CoV spike protein and prevents virus entrance into host cells [10]. In our previous study [10], plant derived secondary metabolite pedalitin has binding energy of –13.832 kcal/mol against RdRp enzyme of SARS-CoV-2 and formed conventional hydrogen bonds with Tyr619 and Thr556 residues. It has been reported that the quinoxaline derivatives have excellent binding configuration and affinity as well as stability with HIV reverse transcriptase enzyme [47]. These results are comparable with the current findings for the binding interaction of aspulvinone D with RdRp stability of HIV-1 and Ebola virus, as well as stability of the complex in dynamic simulation studies.

Various plants and phytochemicals have anti-HIV potential. Gallic acid and ellagic acid have anti-HIV properties and may operate as post-entry inhibitors by decreasing HIV protease activity [48]. *In vitro*, hydroxytyrosol inhibited the viral integrase enzyme and the fusion of the viral envelope with host cells [49]. Apigenin, together with epicatechin, (-)-epicatechin-3-O-gallate (ECG), and epigallocatechin-3-gallate (EGCG), have antiviral properties against HIV, likely through blocking the HIV-1 protease enzyme and reverse transcriptase [45, 46, 49]. In addition, studies have examined the antiviral action of several secondary metabolites against the hepatitis C virus (HCV), that is one of the leading causes of chronic liver disease [45, 50]. Caffeic acid and the flavone apigenin have been shown to impede HCV replication, whilst quercetin-3-O-rutinoside (rutin), curcumin, epigallocatechin-3-gallate (EGCG), and gallic acid have been shown to hinder HCV entrance [51–53]. Different alkaloids extracted from *Atropa belladonna* have been studied for the treatment of EBOLA virus. There is no single drug that is universally accepted for treating dengue fever. However, extracts obtained from various plants are expected to reduce the severity of dengue viruses by increasing platelet count, including *Carica papaya* (papaya) leaf extract used by the local people of India to accelerate the healing process [54, 55]. Some clinical research has shown that giving *C*. *papaya* leaf extract to patients with dengue fever boosts their platelets count [56].

Molecular dynamics simulation is a computational method used to analyze the dynamic interactions within complex systems comprising atoms and molecules over time [57]. Structural parameters such as Root Mean Square Deviation (RMSD), Root Mean Square Fluctuation (RMSF), and the number of intermolecular hydrogen bonds are commonly employed to evaluate the stability, dynamic behavior, and compactness of protein-drug complexes. Specifically, RMSD of the protein backbone serves as a key indicator of stability, with lower values indicating a more stable complex. In this study, we utilized RMSD to assess the stability of RdRp in conjunction with specific phytochemicals, comparing it to the RMSD of the free protein. Mean RMSD values for both free RdRp and RdRp in complex with the mentioned compounds were quantified. Our results indicate that the phytochemicals did not significantly perturb the structural stability of these enzymes. Moreover, molecular dynamics simulation study assists as a vital computational instrument to examine the different chemical, physical and biological processes at molecular level. The finding of this analysis is aligned with a previously reported study [39].

Hydrogen bonds play a crucial role in the formation of secondary and tertiary structural protein motifs [58]. The binding affinity of a drug towards a protein target in molecular dynamics simulations can be explained by the formation of H-bonds between a ligand and a protein motif, where a higher number of H-bonds indicates stronger interactions. In the case of RdRp, cytochalasin Z8, aspulvinone D, and talaromyolide D exhibited hydrogen bonds with receptor proteins. The high affinities of the ligands to RdRp and the stability of these complexes over time can be attributed to the formation of hydrogen bonds. Small non-covalent or reversible covalent inhibitors, like phytochemicals, are generally preferred due to their advantages in terms of side effects and toxicity when compared to covalent inhibitors.

The current study delivers valuable insights into the antiviral potential of secondary metabolites derived as inhibitors of RNA-dependent RNA polymerase via computational docking and MD simulation methods. Additional validation of the results by *in vitro* assays is necessary to verify the effectiveness and specificity of these drug candidates against RdRp. Moreover, potential challenges including off-target effects, toxicity, and bioavailability needs to be explored via experimental validation. In-depth *in vitro* as well as *in vivo* analysis should be carried out to further validate the *in silico* findings, and evaluate the safety and therapeutic potential of selected metabolites in future.

## Conclusion

The ongoing COVID-19 outbreak, and other viral diseases encounter a tremendous effect on the world economy. This study aimed to identify potential inhibitors against key viral enzyme RNA-dependent RNA polymerase. We screened 200 extracellular secondary metabolites from plants and microbes known for their potency against various viral protein receptors. Top five secondary metabolites were screened out with consideration against the RNA-dependent RNA polymerase of SARS-CoV-2, HIV, hepatitis C, Ebola and dengue virus according to hydrophobic associations, binding amino acid residues and lowest binding affinities. Cytochalasin Z8 had the lowest docking score of –8.9 (kcal/mol) against RdRp of SARS-CoV-2 interacted with the binding pocket residues. In this study, aspulvinone D and talaromyolide D demonstrated broad-spectrum antiviral potential against multiple viruses. Aspulvinone D showed the lowest binding affinity with the RNA-dependent RNA polymerase (RdRp) of HIV-1 and Ebola virus, with docking scores of –9.2 and –9.9 kcal/mol, respectively. Talaromyolide D showed interaction with RdRp of hepatitis C and dengue virus, with docking scores of –9.9 and –9.2 kcal/mol, respectively. Drug-behavior and ADMET profiling displayed that selected natural secondary metabolites were found to be non-toxic and non-carcinogenic and possess strong antiviral effects against viral RNA-dependent RNA polymerase enzyme. Consequently, these compounds may be taken into account for further experimental trials and investigations to explore their antiviral potential to treat viral infections in future.

## Supporting information

S1 File S1 Table. Computation binding energy profiling of secondary metabolites as potential drug candidates against RdRp of five different viruses. S2 Table. Computation binding energy profiling of standard FDA proved drug candidates as control against RdRp of five different viruses. S1 Fig. Molecular docking of RdRp of SARS-CoV-2 (7B3B) with Imatinib interactions (A) binding pattern (B), Vorapaxar interactions (C) binding pattern (D), Limonin interactions (E) binding pattern (F) and Isocochliodinol interactions (G) binding pattern (H). S2 Fig. Molecular docking of RdRp of HIV-1 (6UK0) with Isocochliodinol interactions (A) binding pattern (B), Vorapaxar interactions (C) binding pattern (D), Cytochalasin Z8 interactions (E) binding pattern (F) and Imatinib interactions (G) binding pattern (H). S3 Fig. Molecular docking of RdRp of Hepatitis C (4OOW) with Imatinib interactions (A) binding pattern (B), Limonin interactions (C) binding pattern (D), Vorapaxar interactions (E) binding pattern (F) and Raistrickindole A interactions (G) binding pattern (H). S4 Fig. Molecular docking of RdRp of Ebola (7YER) with Corosolic acid interactions (A) binding pattern (B), Limonin interactions (C) binding pattern (D), Cytochalasin Z8 interactions (E) binding pattern (F) and Isocochliodinol interactions (G) binding pattern (H). S5 Fig. Molecular docking of RdRp of Dengue (5K5M) with Isocochliodinol interactions (A) binding pattern (B), Imatinib interactions (C) binding pattern (D), Digitogenin interactions (E) binding pattern (F) and Aspulvinone D interactions (G) binding pattern (H). S6 Fig. Docking of RdRp of SARS-CoV-2 (7B3B) with Remdesivir interaction (A) binding pattern (B), Sofosbuvir interactions (C) binding pattern (D) and Abacavir interactions (E) binding pattern (F). S7 Fig. Docking of RdRp of HIV-1 (6UK0) with Remdesivir interaction (A) binding pattern (B), Sofosbuvir interactions (C) binding pattern (D) and Abacavir interactions (E) binding pattern (F). S8 Fig. Docking of RdRp of Hepatitis C (4OOW) with Remdesivir interaction (A) binding pattern (B), Sofosbuvir interactions (C) binding pattern (D) and Abacavir interactions (E) binding pattern (F). S9 Fig. Docking of RdRp of Ebola (7YER) with Remdesivir interaction (A) binding pattern (B), Sofosbuvir interactions (C) binding pattern (D) and Abacavir interactions (E) binding pattern (F). S10 Fig. Docking of RdRp of Dengue (5K5M) with Remdesivir interaction (A) binding pattern (B), Sofosbuvir interactions (C) binding pattern (D) and Abacavir interactions (E) binding pattern (F). S11 Fig. Ramachandran plot of RdRp receptor protein structures SARS-CoV-2 (A), HIV-1 (B), Hepatitis C (C), Ebola (D), and Dengue (E) analyzed by PROCHECK online web tool. S12 Fig. First pose 2D images of MD trajectories of the best selected ligands and remdesivir as a control drug. S13 Fig. Last pose 2D images of MD trajectories of the best selected ligands and remdesivir as a control drug.
(DOCX)
