# Supplementary material for: Structure based screening and molecular docking with dynamic simulation of natural secondary metabolites to target RNA-dependent RNA polymerase of five different retroviruses
Source: PLoS One. 2024 Aug 5;19(8):e0307615. doi: 10.1371/journal.pone.0307615 (PMC11299834; doi:10.1371/journal.pone.0307615)
Supplement: S1 File — S1 Table. Computation binding energy profiling of secondary metabolites as potential drug candidates against RdRp of five different viruses. S2 Table. Computation binding energy profiling of standard FDA proved drug candidates as control against RdRp of five different viruses. S1 Fig. Molecular docking of RdRp of SARS-CoV-2 (7B3B) with Imatinib interactions (A) binding pattern (B), Vorapaxar interactions (C) binding pattern (D), Limonin interactions (E) binding pattern (F) and Isocochliodinol interactions (G) binding pattern (H). S2 Fig. Molecular docking of RdRp of HIV-1 (6UK0) with Isocochliodinol interactions (A) binding pattern (B), Vorapaxar interactions (C) binding pattern (D), Cytochalasin Z8 interactions (E) binding pattern (F) and Imatinib interactions (G) binding pattern (H). S3 Fig. Molecular docking of RdRp of Hepatitis C (4OOW) with Imatinib interactions (A) binding pattern (B), Limonin interactions (C) binding pattern (D), Vorapaxar interactions (E) binding pattern (F) and Raistrickindole A interactions (G) binding pattern (H). S4 Fig. Molecular docking of RdRp of Ebola (7YER) with Corosolic acid interactions (A) binding pattern (B), Limonin interactions (C) binding pattern (D), Cytochalasin Z8 interactions (E) binding pattern (F) and Isocochliodinol interactions (G) binding pattern (H). S5 Fig. Molecular docking of RdRp of Dengue (5K5M) with Isocochliodinol interactions (A) binding pattern (B), Imatinib interactions (C) binding pattern (D), Digitogenin interactions (E) binding pattern (F) and Aspulvinone D interactions (G) binding pattern (H). S6 Fig. Docking of RdRp of SARS-CoV-2 (7B3B) with Remdesivir interaction (A) binding pattern (B), Sofosbuvir interactions (C) binding pattern (D) and Abacavir interactions (E) binding pattern (F). S7 Fig. Docking of RdRp of HIV-1 (6UK0) with Remdesivir interaction (A) binding pattern (B), Sofosbuvir interactions (C) binding pattern (D) and Abacavir interactions (E) binding pattern (F). S8 Fig. Docking of RdRp of [file pone.0307615.s001.docx]

Structure based Screening and Molecular Docking with Dynamic Simulation of Natural Secondary Metabolites to Target RNA-dependent RNA polymerase of Five Different Retroviruses

**Muhammad Azeem^1^, Ghulam Mustafa^2^*, Sibtain Ahmed^3^, Amna Mushtaq^4^, Muhammad Arshad^5^, Muhammad Usama^2^ and Muhammad Farooq^2^**

^1^College of Life Sciences, Anhui Normal University, Wuhu 241000, China

^2^Department of Biochemistry, Government College University Faisalabad, Faisalabad, 38000 Pakistan

^3^Department of Biochemistry, Bahauddin Zakariya University, Multan-60800, Pakistan

^4^Department of Medical Laboratory, TIMES Institute, Multan, Pakistan

^5^Department of Basic Sciences, University of Veterinary and Animal Sciences, Jhang-Campus, Lahore, Pakistan

*Correspondence: [drghulammustafa@gcuf.edu.pk](mailto:drghulammustafa@gcuf.edu.pk)

# Supplementary Materials

**Table S1.** Computation binding energy profiling of secondary metabolites as potential drug candidates against RdRp of five different viruses.

| RdRp | Ligand | PubChem ID | Binding Affinity (kcal/mol) | Residues |
| --- | --- | --- | --- | --- |
| SARS-CoV-2 (7B3B) | Imatinib | 5291 | -9.4 | ProA:461, ArgA:349, ProA:323, SerA:318, ProA:677, SerA:255, ProA:322, TyrA:255, TrpA:268, IleA:266 |
|  | Vorapaxar | 10077130 | -9.2 | ThrA:141, AsnA:138, TyrA:32, LysA:47, TyrA:129, AsnA:781, AlaA:706 |
|  | Limonin | 179651 | -9.1 | AspA:618, SerA:814, CysA:813, LysA:798, SerA:549, AlaA:547, AgrA:555, LysA:551 |
|  | Isocochliodinol | 474301 | -9.1 | AlaA:176, AlaA:253, LeuA:261, ThrA:319, SerA:255, TrpA:268, IleA:266, LeuA:270, ProA:322, TyrA:265 |
| HIV-1 (6UK0) | Isocochliodinol | 474301 | -9.4 | HisA:96, TrpA:266, ValA:276, LysA:353, TyrA:354, AlaA:355, ArgA:356, LysA:374 |
|  | Vorapaxar | 10077130 | -9.1 | ProA:345, GluA:344, TyrA:342, IleA:309, ProA:272, ValA:314, HisA:315 |
|  | Cytochalasin Z8 | 11518356 | -8.9 | ValA:90, GlnA:182, ArgA:172, IleA:180, ValA:381 |
|  | Imatinib | 5291 | -8.9 | HisA:315, ValA:317, LysA:347, AsnA:348, TheA:346, GlnA:343 |
| Hepatitis C (4OOW) | Imatinib | 5291 | -10.2 | IleA:405, LysA:141, IleA:160, SerA:282, ProA:93, AspA:225, CysA:451, AspA:559 |
|  | Limonin | 179651 | -10 | GlyA:449, CysA:366, SerA:367 |
|  | Vorapaxar | 10077130 | -9.7 | AlaA:97, IleA:160, LysA:141, ArgA:394, ArgA:401 |
|  | Raistrickindole A | 145720909 | -9.7 | IleA:160, PheA:162, ArgA:168, GlyA:557, AspA:559 |
| Ebola (7YER) | Corosolic acid | 6918774 | -10.7 | AspA;290, TrpA:212, SerA:34, HisA:381 |
|  | Limonin | 179651 | -10.6 | AsnA:743, ArgA:561, LysA:803, TyrA:800 |
|  | Cytochalasin Z8 | 11518356 | -10.4 | LysA:803, TyrA:800, ProA:300, LysA:296 |
|  | Isocochliodinol | 474301 | -9.7 | IleA:1112, LeuA:1127, IleA:1128, GlyA:1129, ThrA:1131, PheA:1280, ArgA:1354, ValA:1356 |
| Dengue (5K5M) | Isocochliodinol | 474301 | -9.8 | HisA:798, SerA:601, GluA:485, TheA:486, LysA:402, AspA:539 |
|  | Imatinib | 5291 | -9.6 | ArgA:599, AspA:539, AsnA:610, TyrA:607, ArgA:482, GluA:485, SerA:661 |
|  | Digitogenin | 441886 | -9.4 | TyrA:573, CysA:709, SerA:796, HisA:798 |
|  | Aspulvinone D | 54678424 | -9.2 | GluA:494, LysA:402, ArgA;792, PheA:486, TrpA:795, PheA:399 |

| **(A)** | **(B)** |
| --- | --- |
| 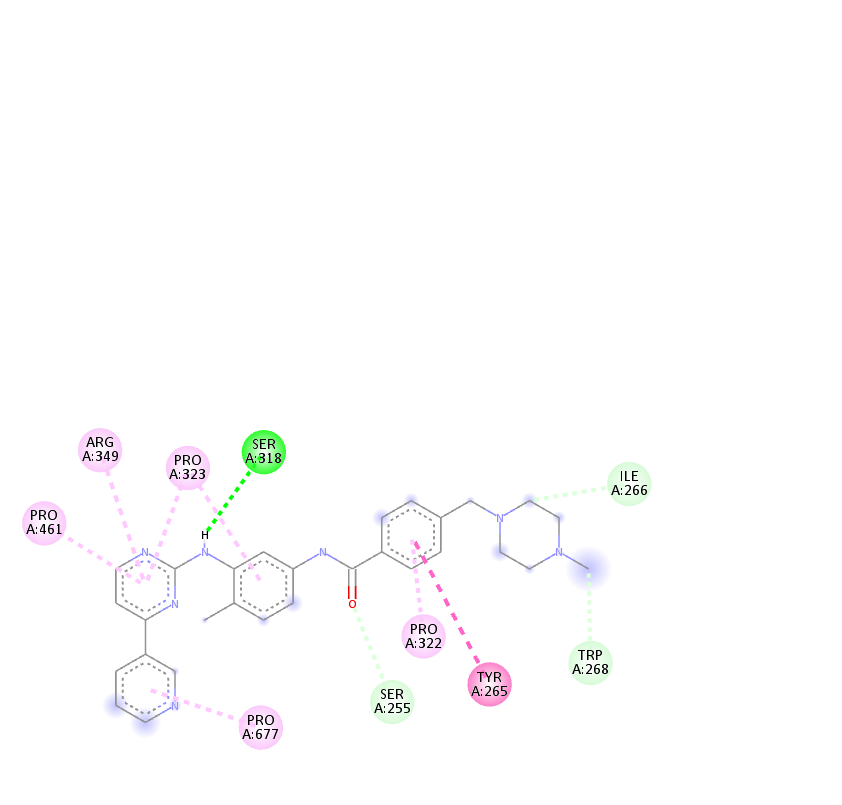 | 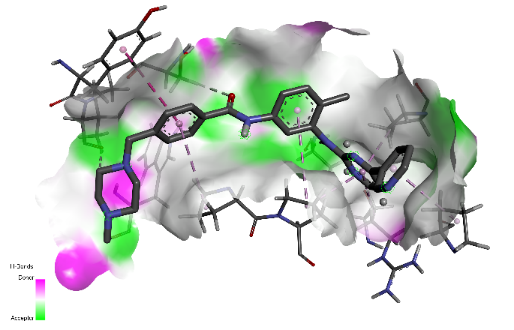 |
| **(C)** | **(D)** |
| 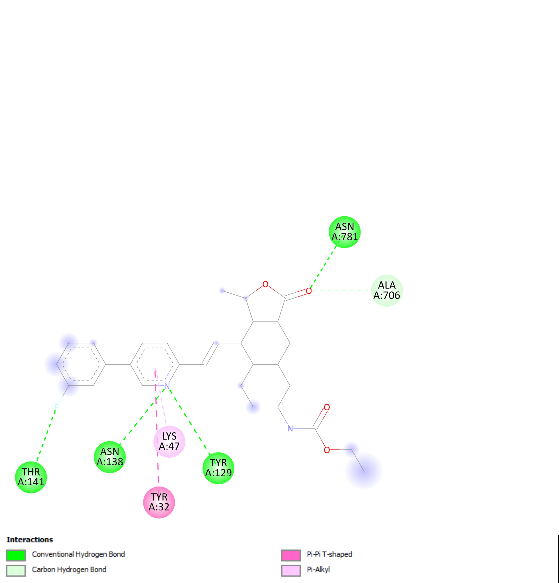 | 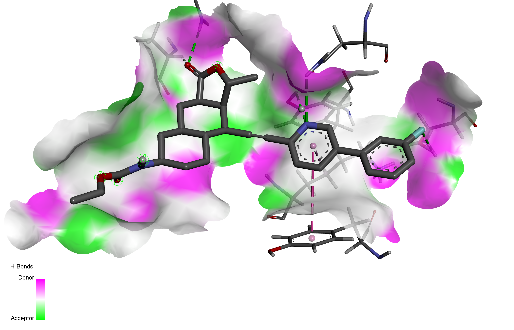 |
| **(E)** | **(F)** |
| 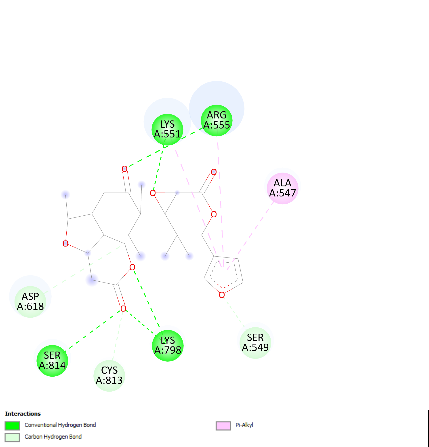 | **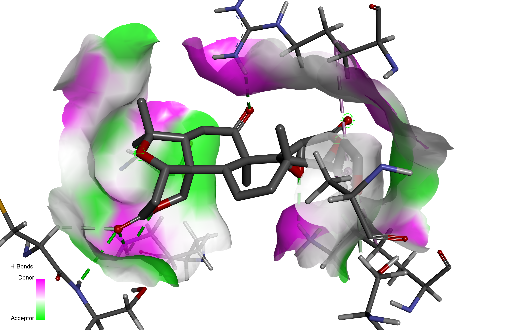** |
| **(G)** | **(H)** |
| 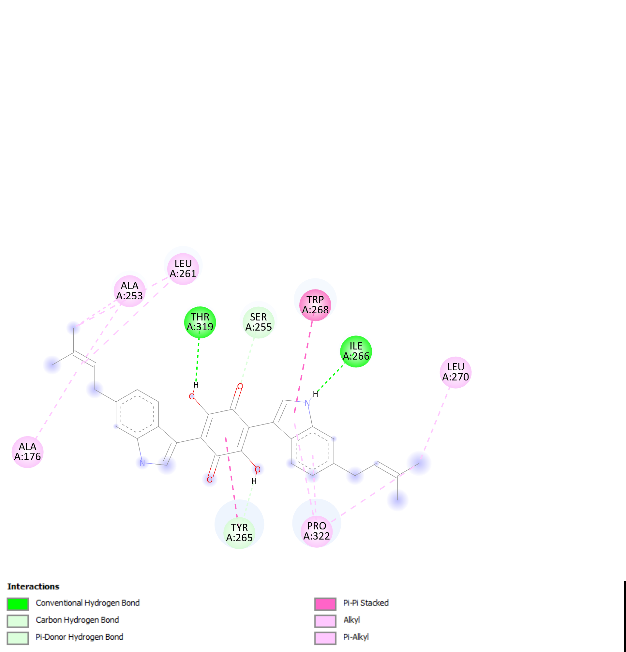 | 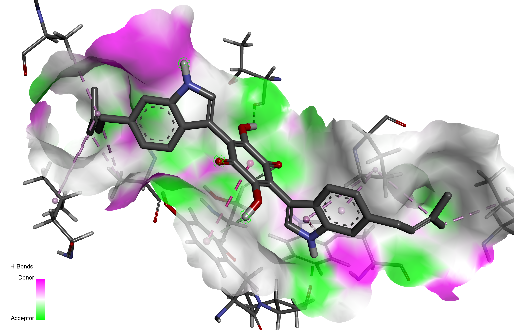 |

Figure S1. Molecular docking of RdRp of SARS-CoV-2 (7B3B) with Imatinib interactions (A) binding pattern (B), Vorapaxar interactions (C) binding pattern (D), Limonin interactions (E) binding pattern (F) and Isocochliodinol interactions (G) binding pattern (H).

| **(A)** | **(B)** |
| --- | --- |
| **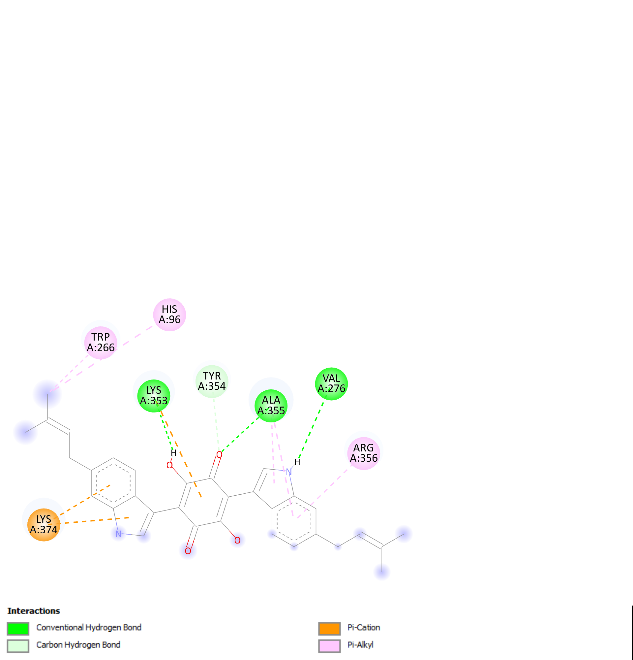** | **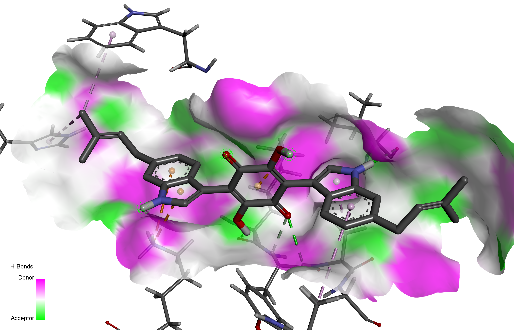** |
| **(C)** | **(D)** |
| 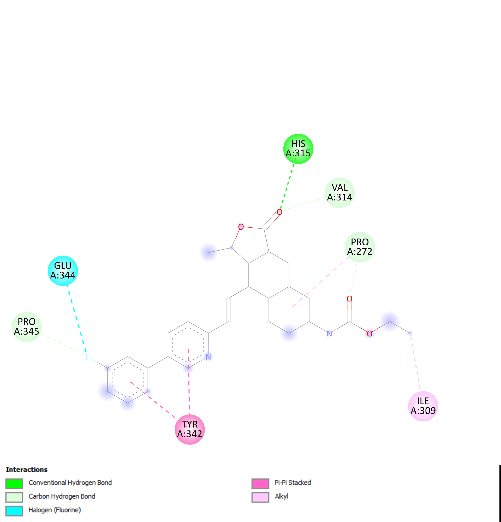 | 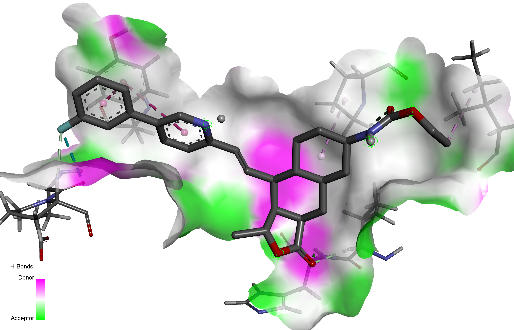 |
| **(E)** | **(F)** |
| **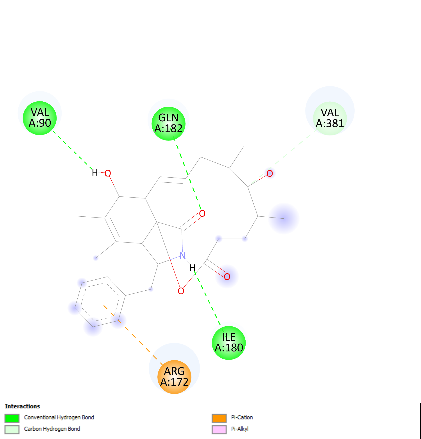** | **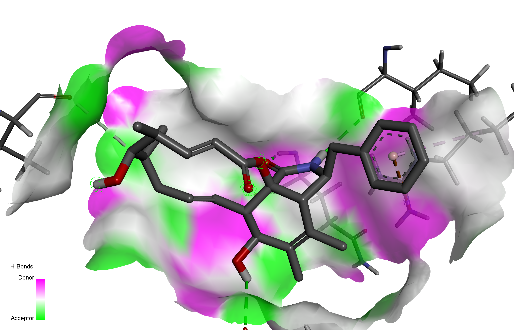** |
| **(G)** | **(H)** |
| **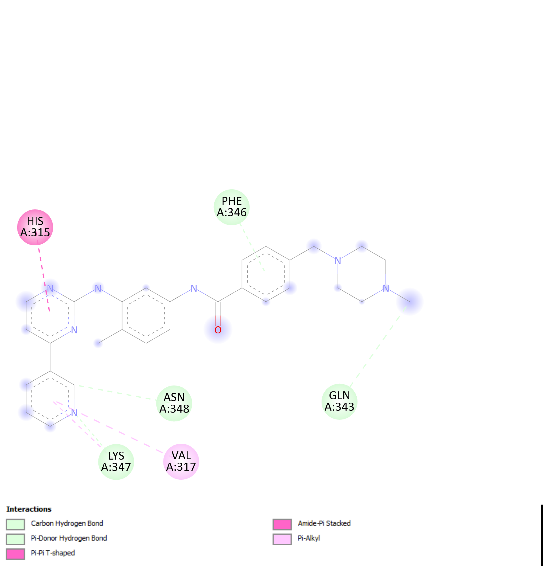** | **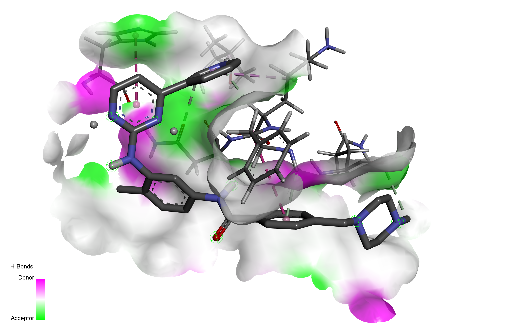** |

Figure S2. Molecular docking of RdRp of HIV-1 (6UK0) with Isocochliodinol interactions (A) binding pattern (B), Vorapaxar interactions (C) binding pattern (D), Cytochalasin Z8 interactions (E) binding pattern (F) and Imatinib interactions (G) binding pattern (H).

| **(A)** | **(B)** |
| --- | --- |
| 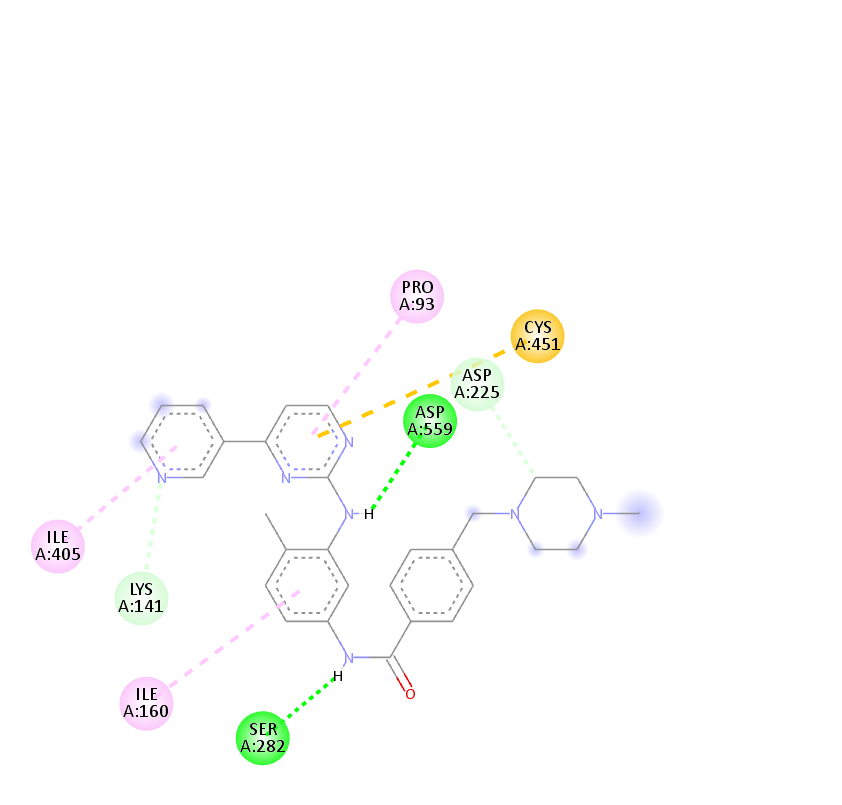 | **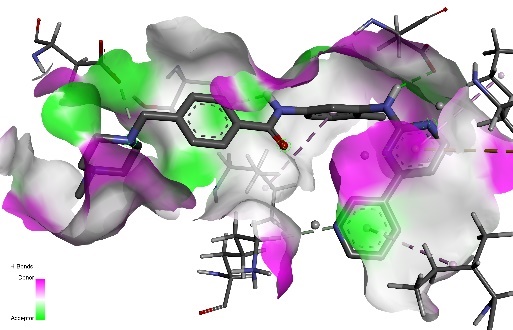** |
| **(C)** | **(D)** |
| 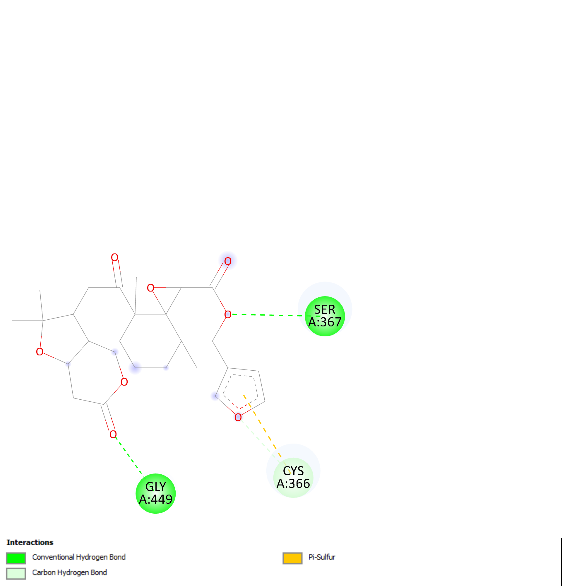 | 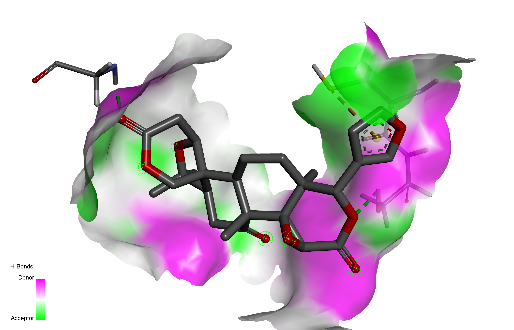 |
| **(E)** | **(F)** |
| **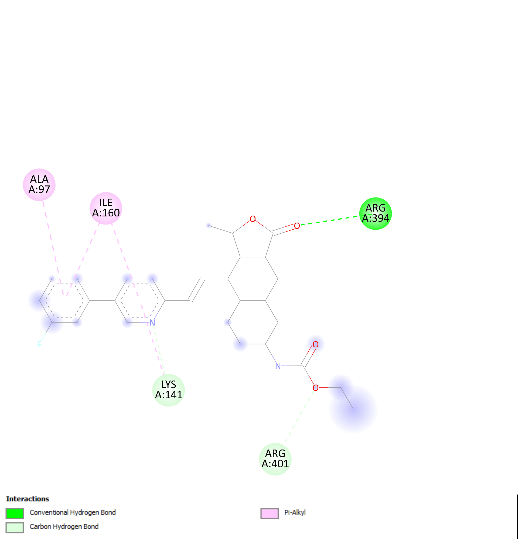** | **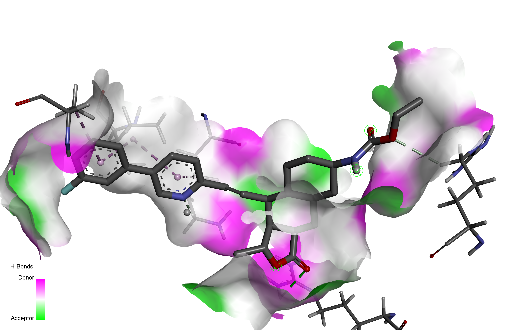** |
| **(G)** | **(H)** |
| **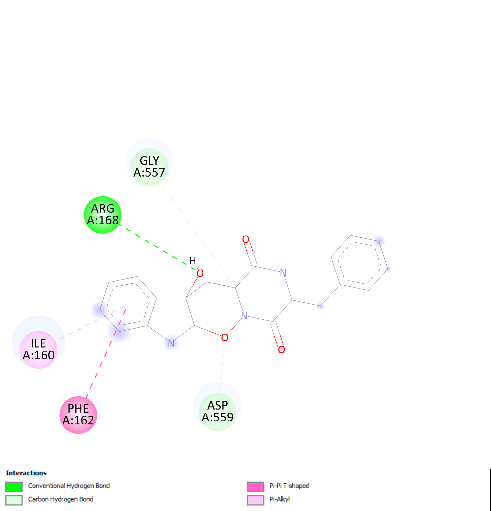** | **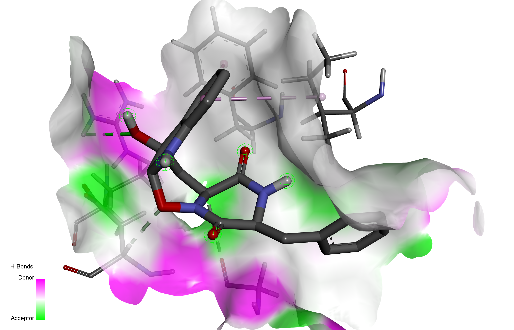** |

Figure S3. Molecular docking of RdRp of Hepatitis C (4OOW) with Imatinib interactions (A) binding pattern (B), Limonin interactions (C) binding pattern (D), Vorapaxar interactions (E) binding pattern (F) and Raistrickindole A interactions (G) binding pattern (H).

| **(A)** | **(B)** |
| --- | --- |
| 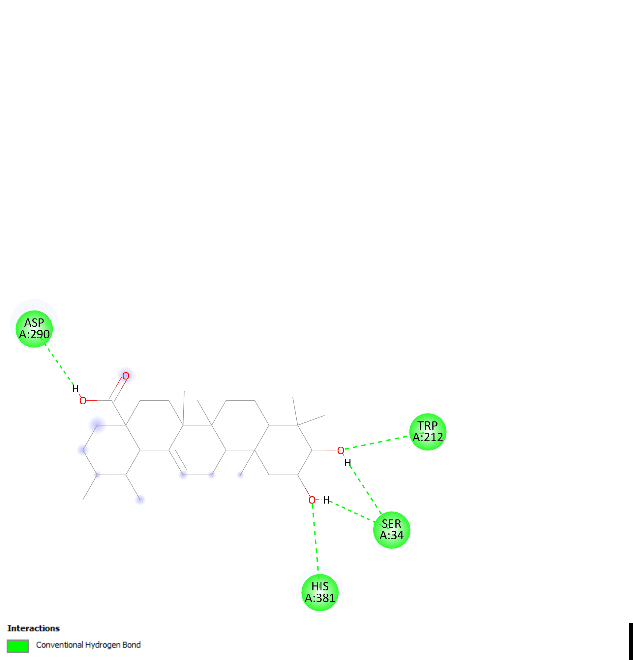 | **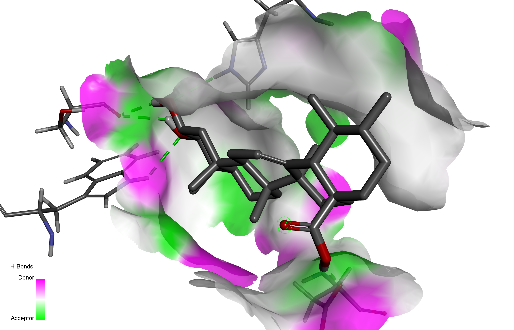** |
| **(C)** | **(D)** |
| 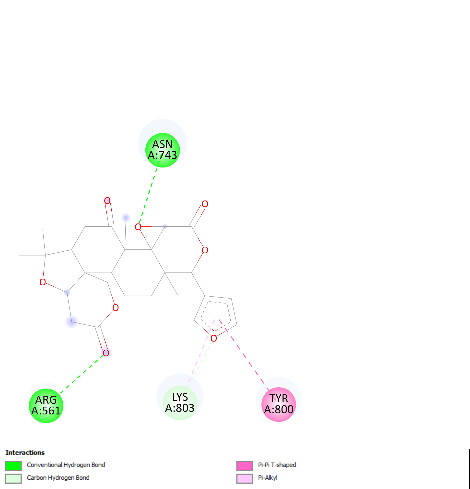 | 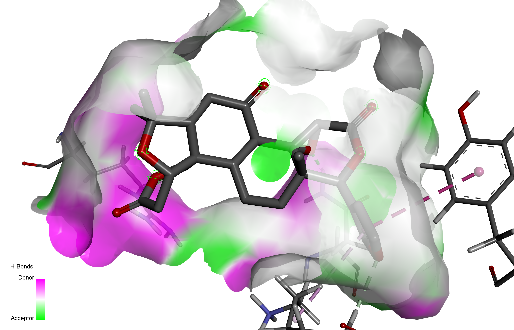 |
| **(E)** | **(F)** |
| **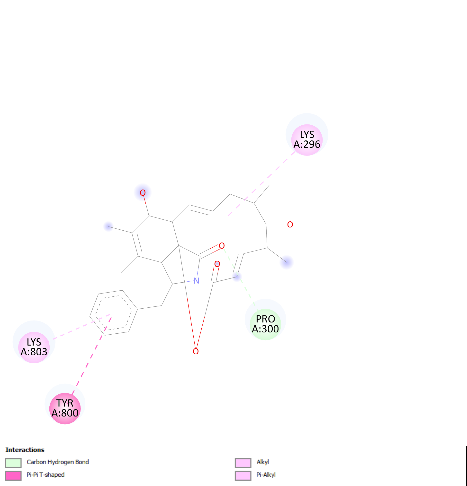** | **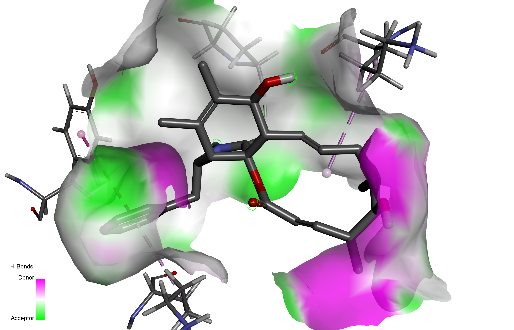** |
| **(G)** | **(H)** |
| **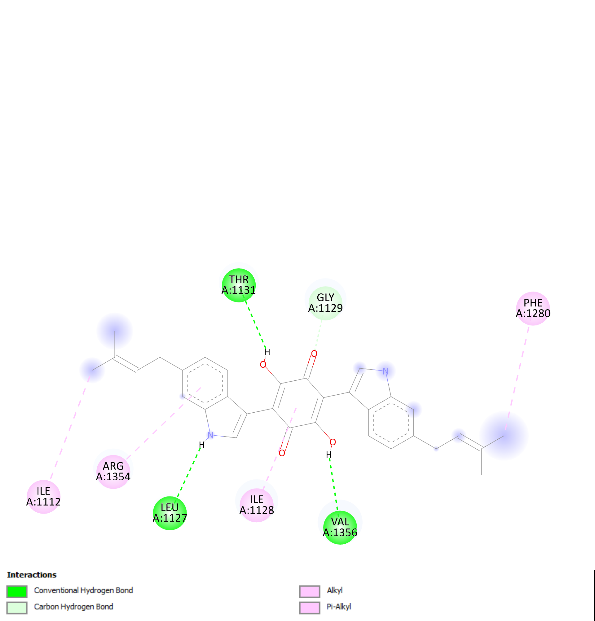** | **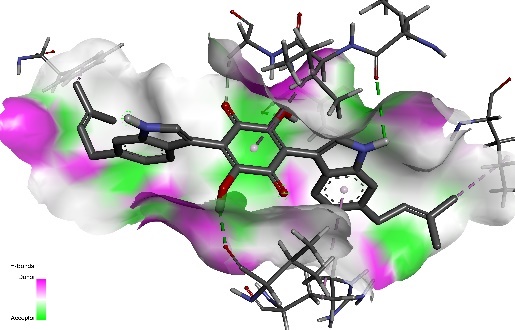** |

Figure S4. Molecular docking of RdRp of Ebola (7YER) with Corosolic acid interactions (A) binding pattern (B), Limonin interactions (C) binding pattern (D), Cytochalasin Z8 interactions (E) binding pattern (F) and Isocochliodinol interactions (G) binding pattern (H).

| **(A)** | **(B)** |
| --- | --- |
| **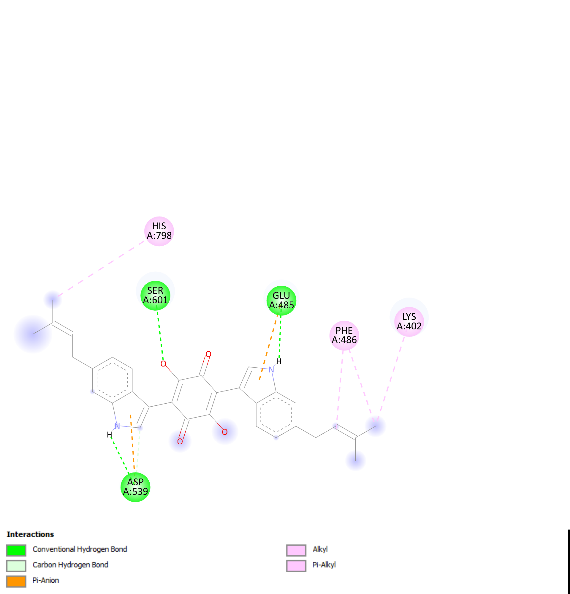** | **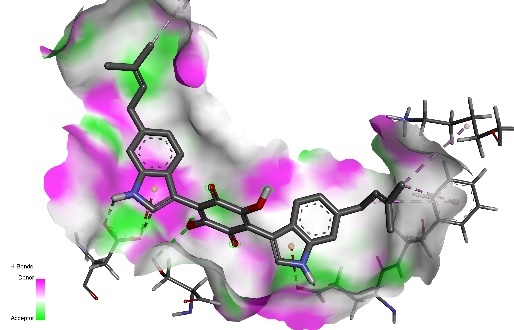** |
| **(C)** | **(D)** |
| 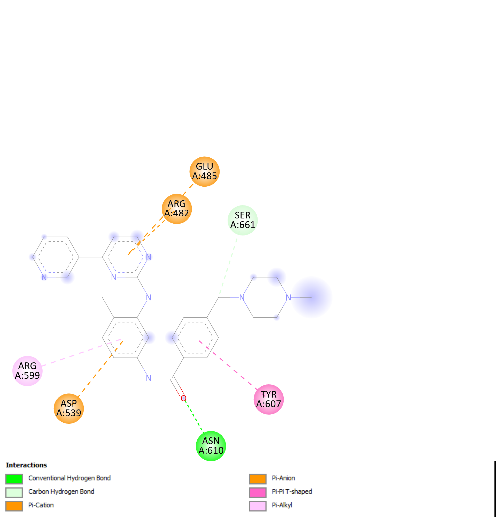 | 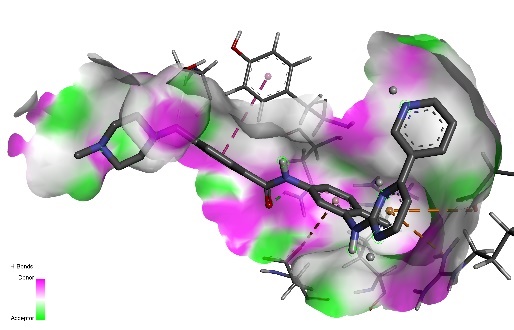 |
| **(E)** | **(F)** |
| **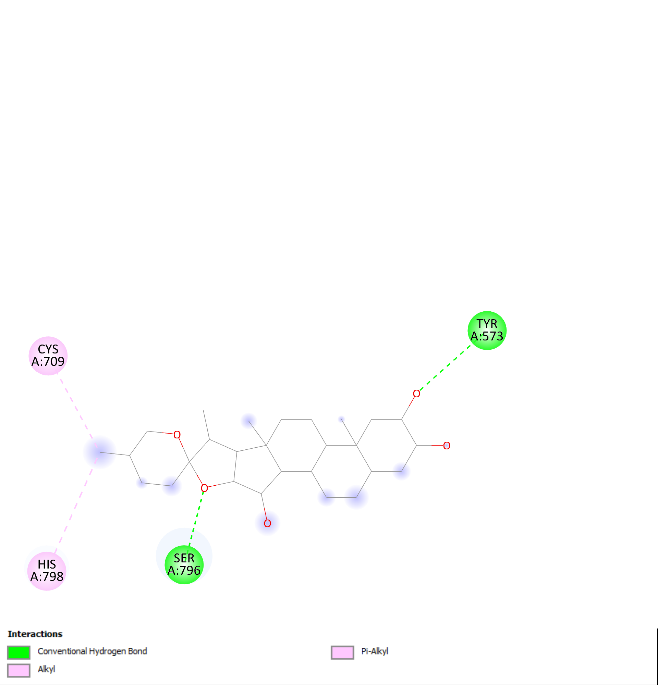** | **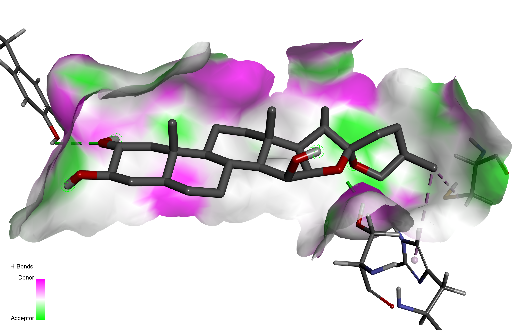** |
| **(G)** | **(H)** |
| **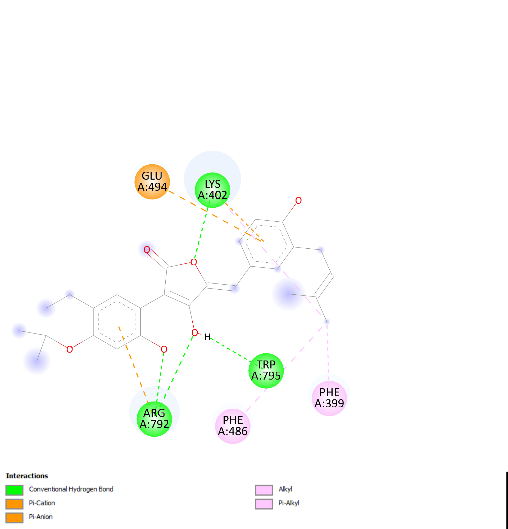** | **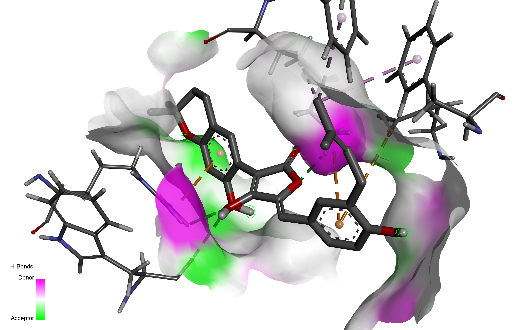** |

Figure S5. Molecular docking of RdRp of Dengue (5K5M) with Isocochliodinol interactions (A) binding pattern (B), Imatinib interactions (C) binding pattern (D), Digitogenin interactions (E) binding pattern (F) and Aspulvinone D interactions (G) binding pattern (H).

**Table S2.** Computation binding energy profiling of standard FDA proved drug candidates as control against RdRp of five different viruses.

| RdRp | Ligand | PubChem ID | Binding Affinity (kcal/mol) | Residues |
| --- | --- | --- | --- | --- |
| SARS-CoV-2 (7B3B) | Sofosbuvir (Control) | 45375808 | -8.3 | AlaA:502, AlaA:512, ValA:560, ThrA:565, AgrA:569, LeuA:576, AlaA:685, ThrA:687, AlaA:688 |
|  | Abacavir (Control) | 441300 | -6.9 | TryA:32, SerA:709, LysA:47, TyrA:129, AsnA:138 |
| HIV-1 (6UK0) | Sofosbuvir (Control) | 45375808 | -7.8 | GlnA:91, ValA:90, IleA:180, TyrA:181, ValA:179, ArgA:172 |
|  | Abacavir (Control) | 441300 | -7.0 | IleA:94, TrpA:266, HisA:96, GlnA:269, LysA:350, LysA:353, AsnA:265, GluA:378 |
| Hepatitis C (4OOW) | Sofosbuvir (Control) | 45375808 | -8.0 | IleA:160, PheA:162, AlaA:97, ProA:93, AspA:559, GlyA:557, GlyA:449, CysA:451 |
|  | Abacavir (Control) | 441300 | -7.6 | LeuA:159, ArgA:158, AspA:225, AspA:318, SerA:282 |
| Ebola (7YER) | Sofosbuvir (Control) | 45375808 | -8.0 | LysA:373, LysA:555, LeuA:557, GluA:556, LysA:296, GlnA:372, ProA:300, LysA:635, AspA:632, GluA:791, PheA:793, AsnA:743, ThrA:631, LeuA:603, PheA:369, PheA:801, LysA:803, TyrA:800, ArgA:561 |
|  | Abacavir (Control) | 441300 | -7.2 | ArgA:338, IleA:337, AspA:290, SerA:289, ProA:72, ValA:379, SerA:34 |
| Dengue (5K5M) | Sofosbuvir (Control) | 45375808 | -7.7 | PheA:486, GlyA:490, ArgA:482, GluA:485, AsnA:610, SerA:601, IleA:797, TyrA:607, LysA:402, GluA:494 |
|  | Abacavir (Control) | 441300 | -7.2 | AlaA:532, AspA;664, CysA:665, ProA:707, AspA;690, AspA:534 |

| **(A)** | **(B)** |
| --- | --- |
| **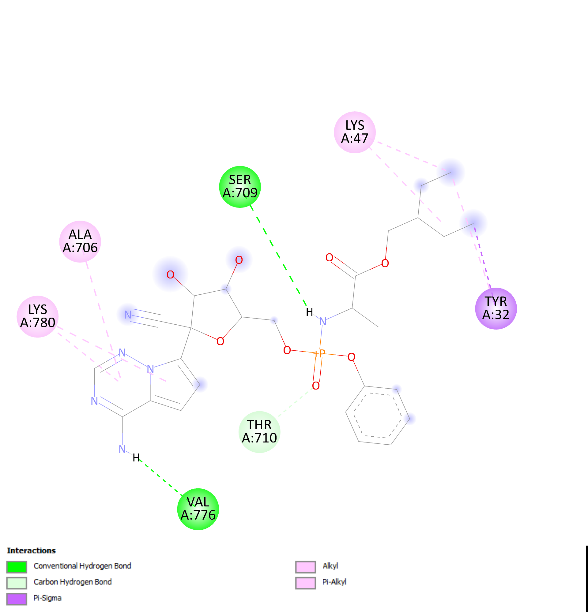** | **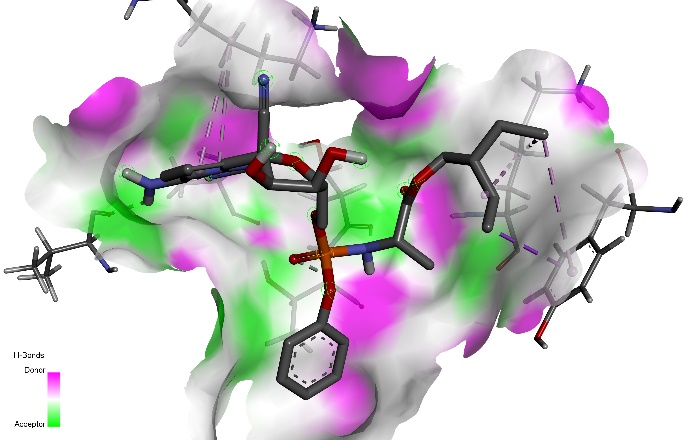** |
| **(C)** | **(D)** |
| **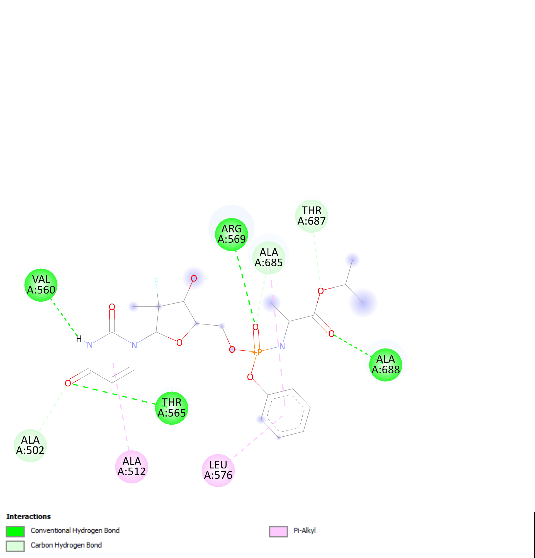** | **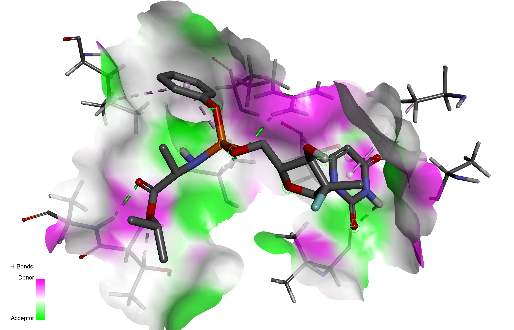** |
| **(E)** | **(F)** |
| 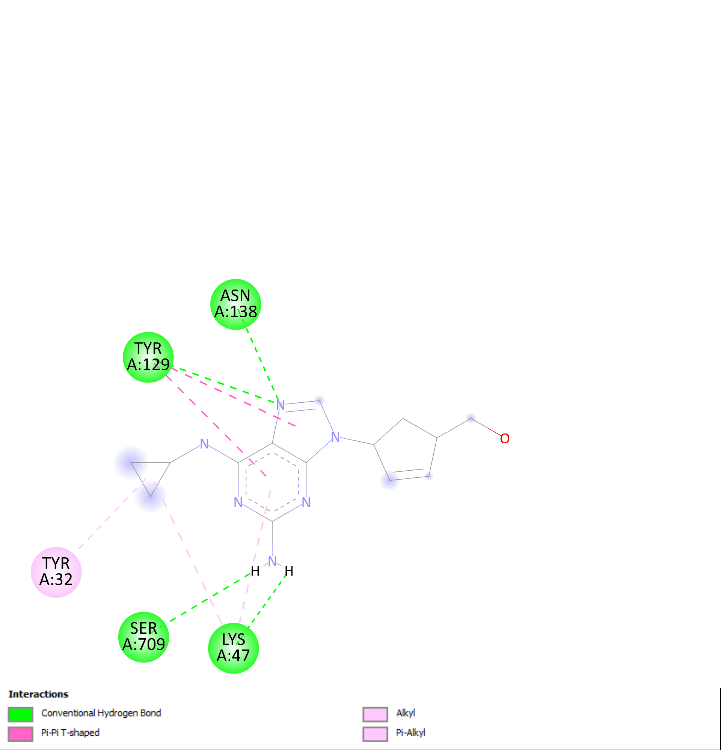 | 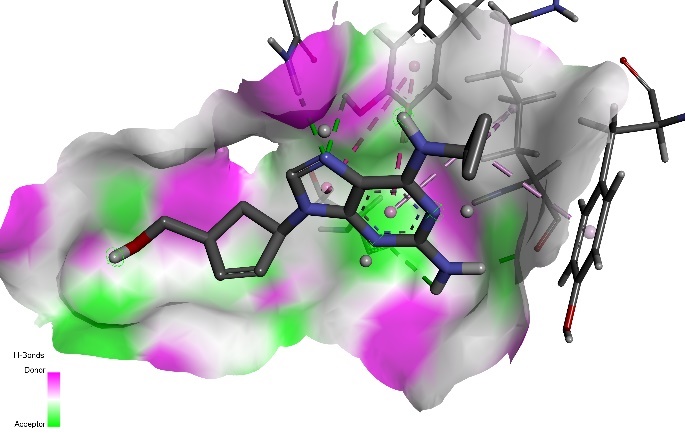 |

Figure S6. Docking of RdRp of SARS-CoV-2 (7B3B) with Remdesivir interaction (A) binding pattern (B), Sofosbuvir interactions (C) binding pattern (D), and Abacavir interactions (E) binding pattern (F).

| **(A)** | **(B)** |
| --- | --- |
| **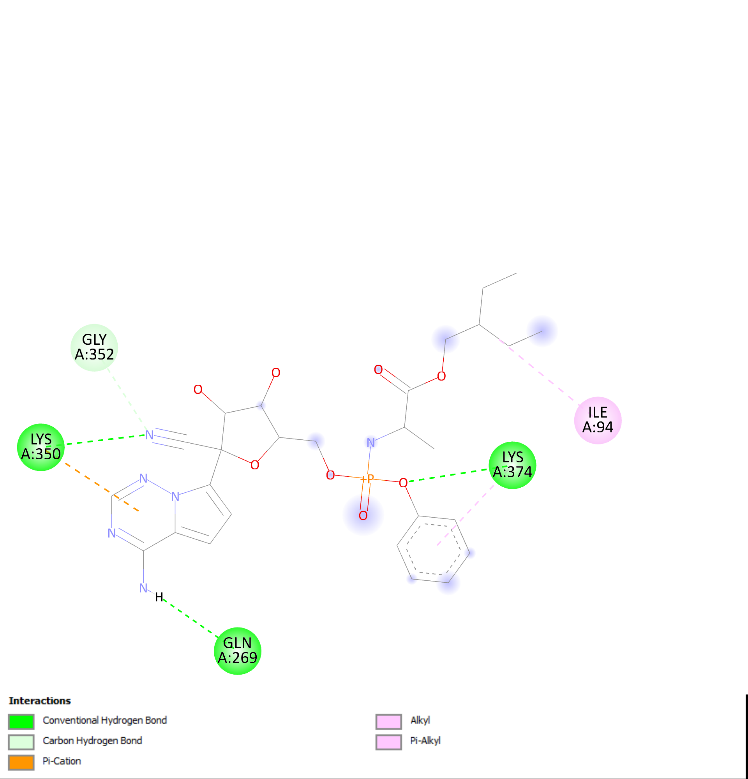** | **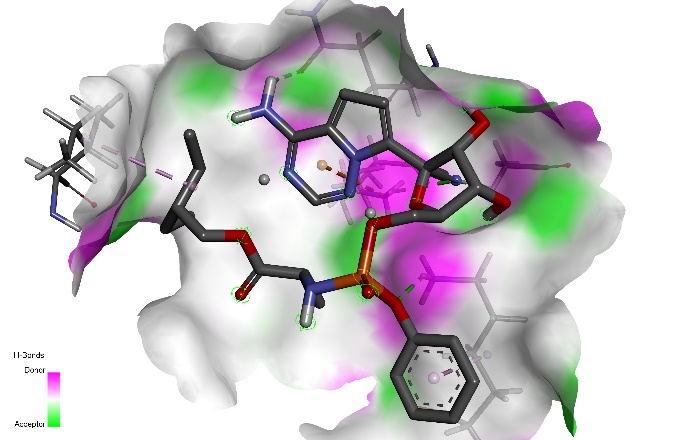** |
| **(C)** | **(D)** |
| **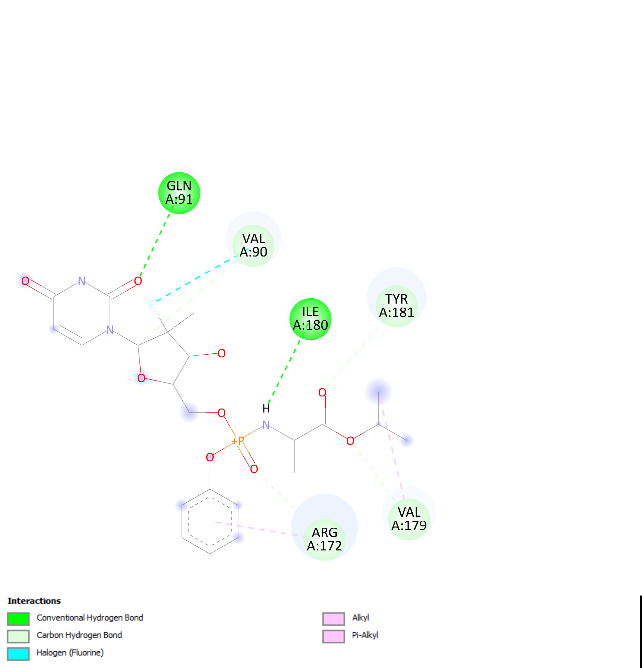** | **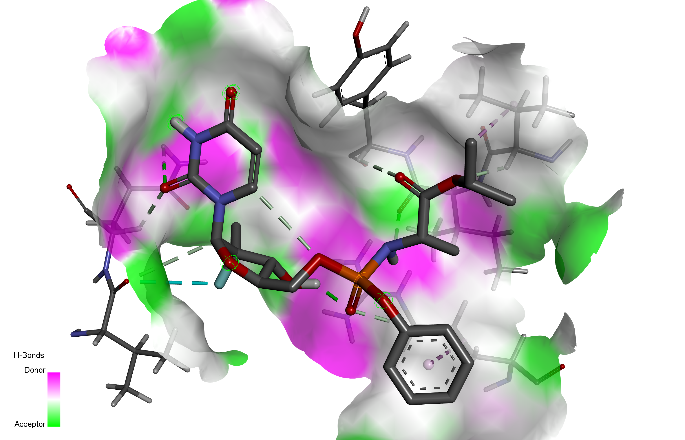** |
| **(E)** | **(F)** |
| 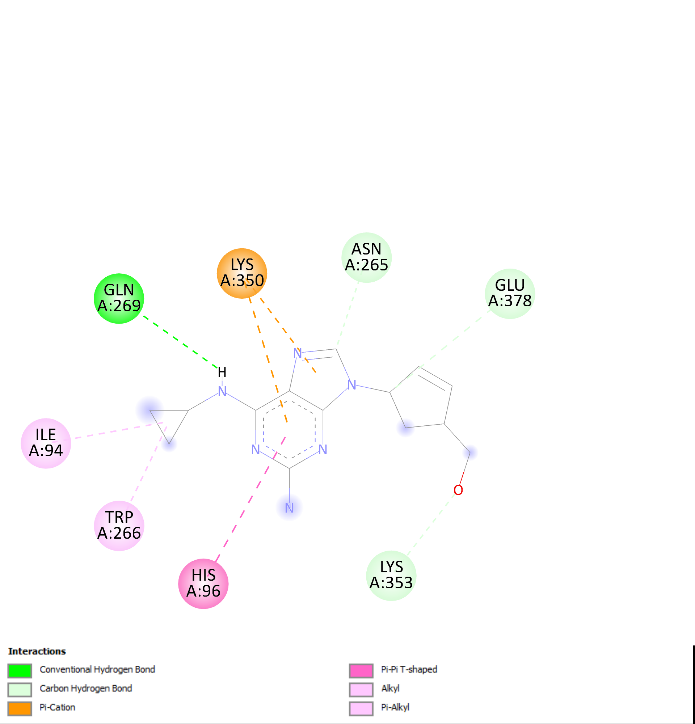 | 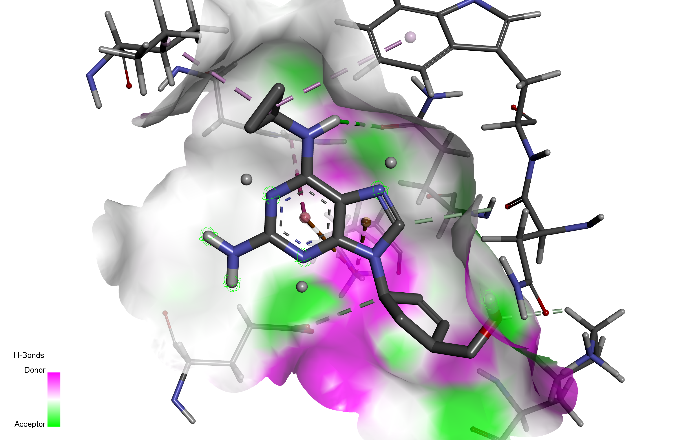 |

Figure S7. Docking of RdRp of HIV-1 (6UK0) with Remdesivir interaction (A) binding pattern (B), Sofosbuvir interactions (C) binding pattern (D), and Abacavir interactions (E) binding pattern (F).

| **(A)** | **(B)** |
| --- | --- |
| **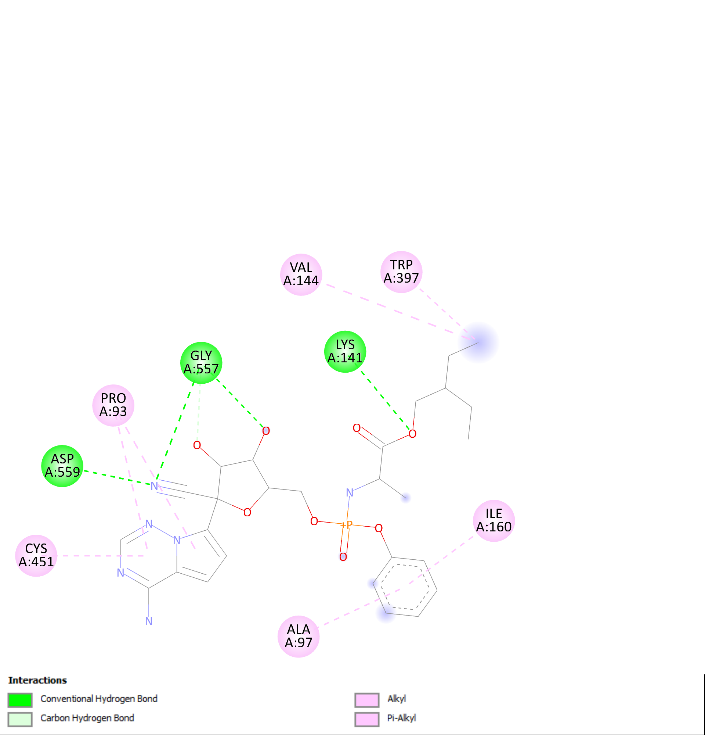** | **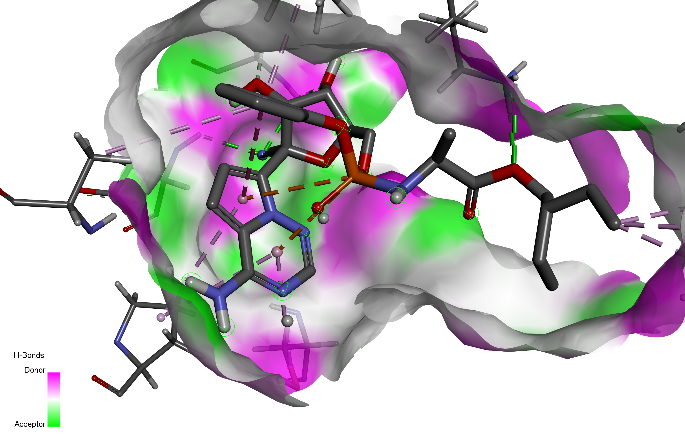** |
| **(C)** | **(D)** |
| **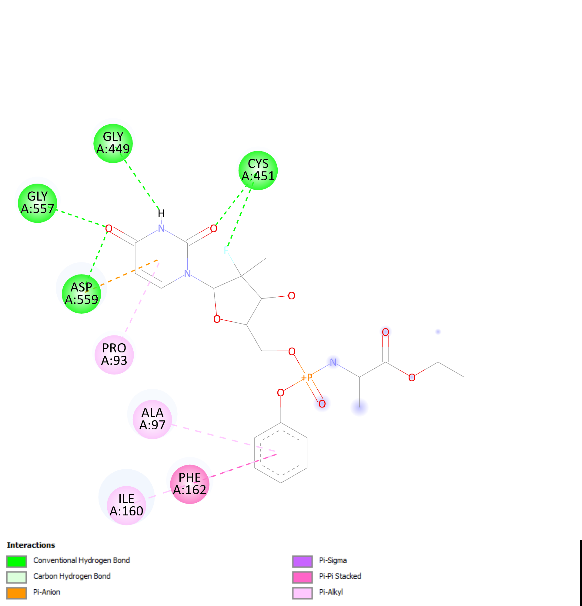** | **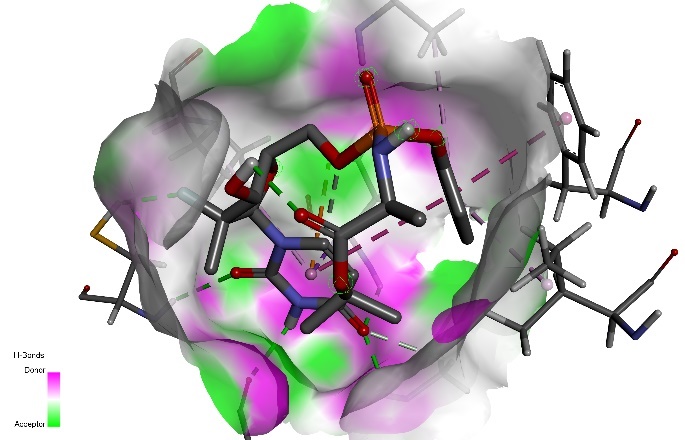** |
| **(E)** | **(F)** |
| 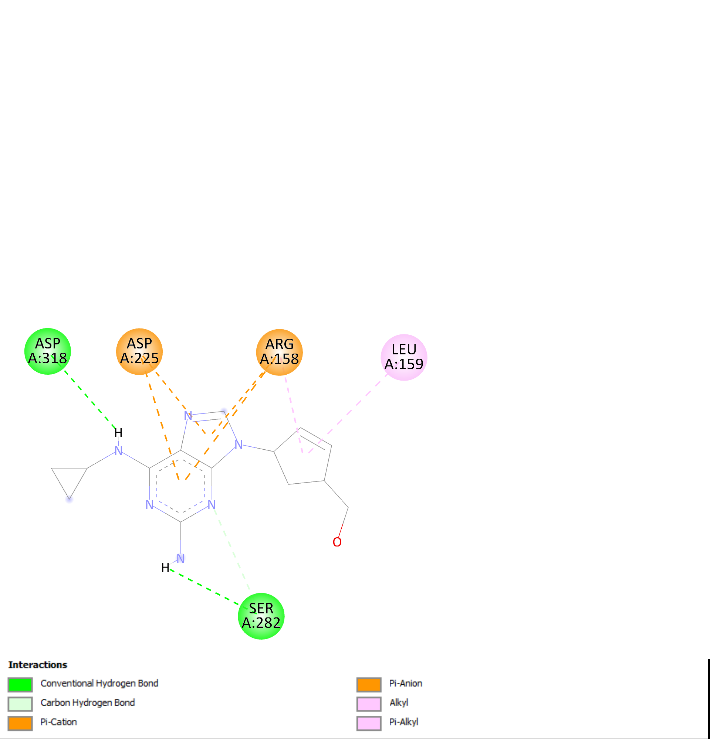 | 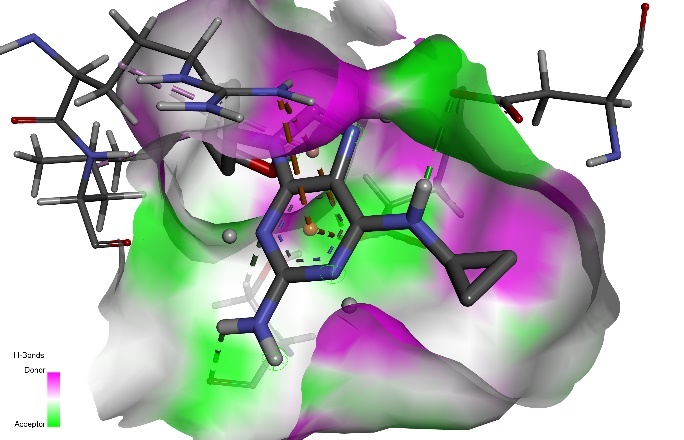 |

Figure S8. Docking of RdRp of Hepatitis C (4OOW) with Remdesivir interaction (A) binding pattern (B), Sofosbuvir interactions (C) binding pattern (D), and Abacavir interactions (E) binding pattern (F).

| **(A)** | **(B)** |
| --- | --- |
| **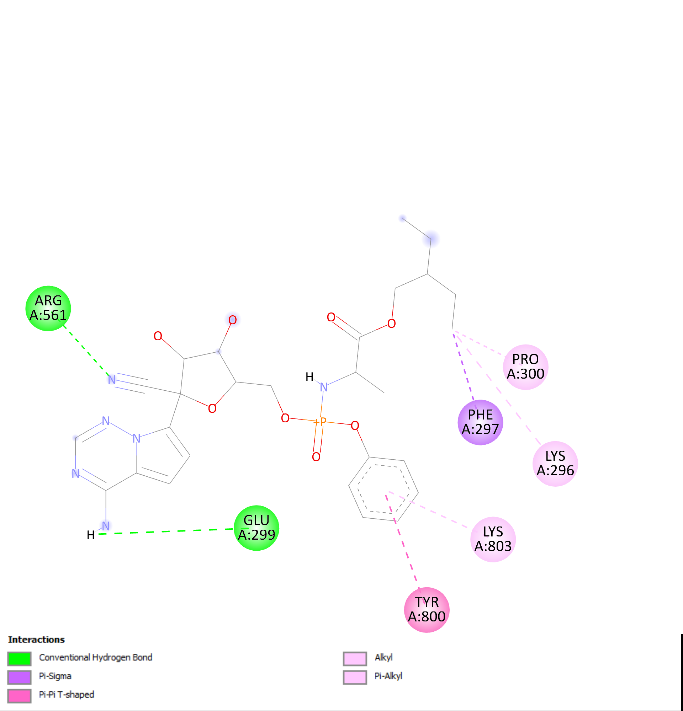** | **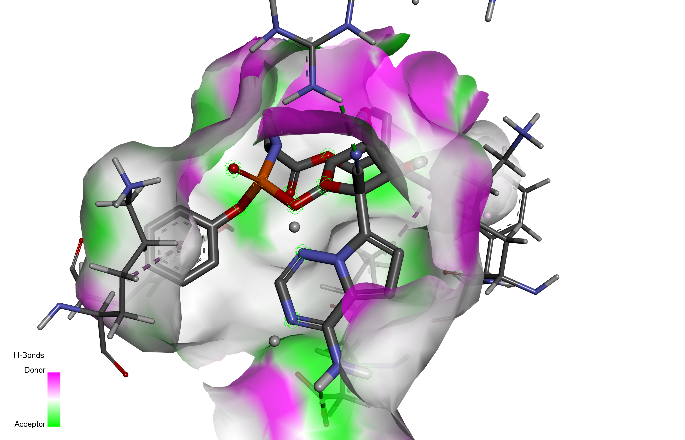** |
| **(C)** | **(D)** |
| **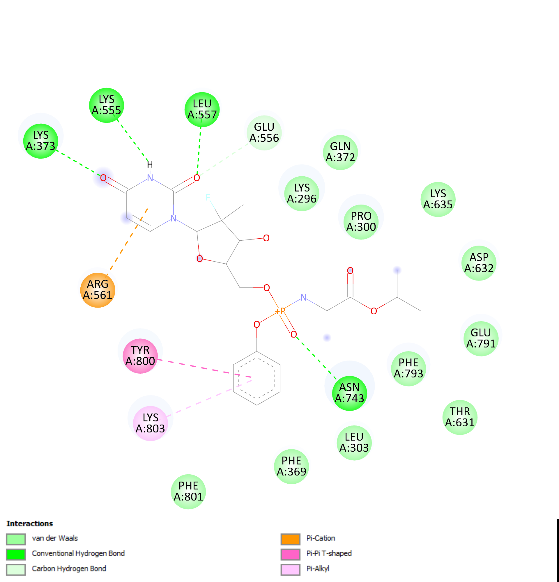** | **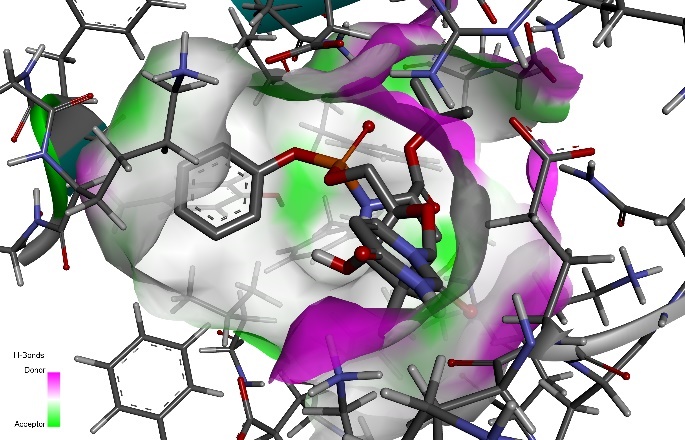** |
| **(E)** | **(F)** |
| 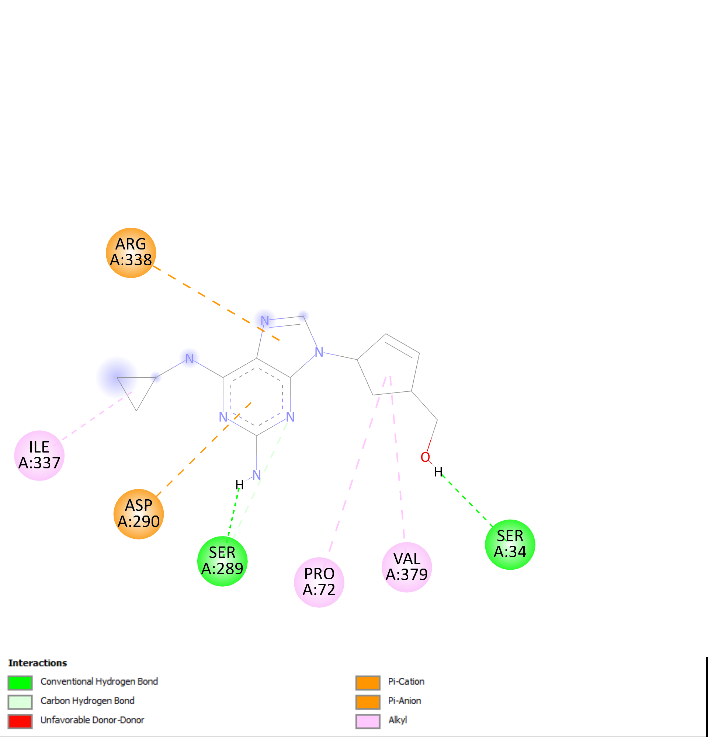 | 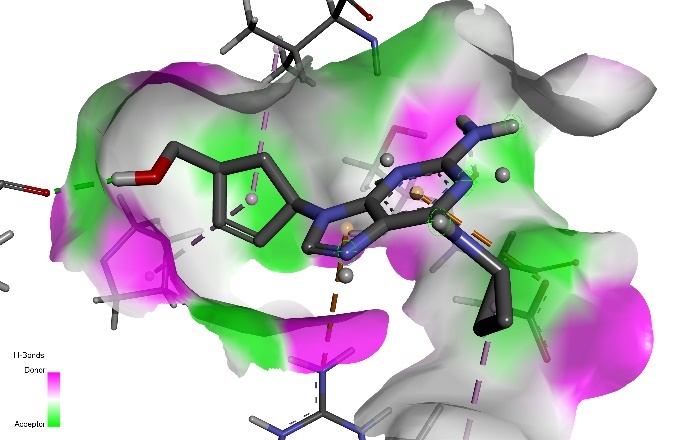 |

Figure S9. Docking of RdRp of Ebola (7YER) with Remdesivir interaction (A) binding pattern (B), Sofosbuvir interactions (C) binding pattern (D), and Abacavir interactions (E) binding pattern (F).

| **(A)** | **(B)** |
| --- | --- |
| **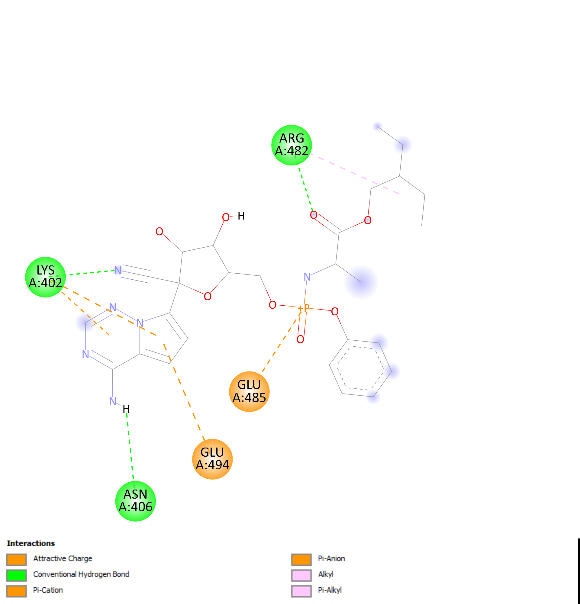** | **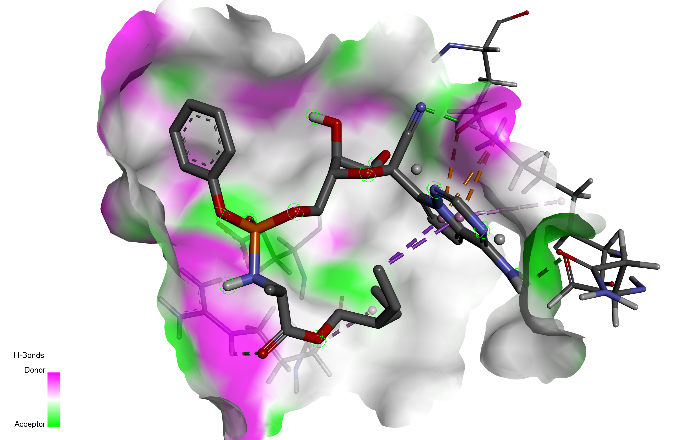** |
| **(C)** | **(D)** |
| **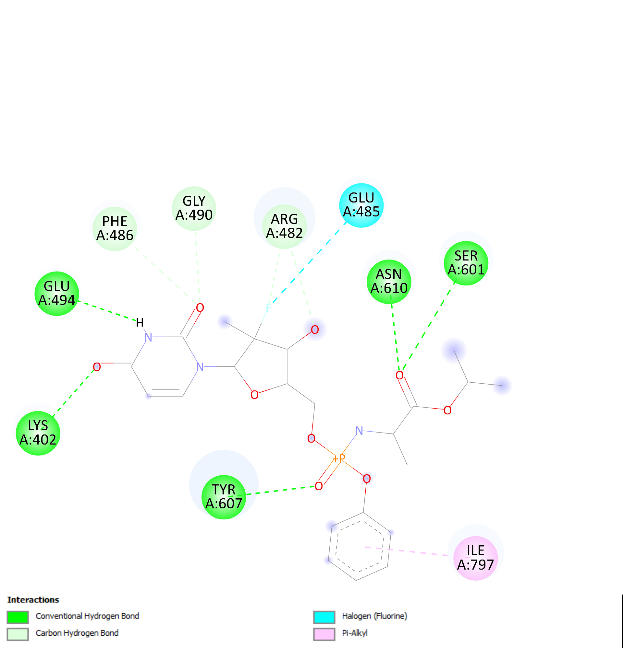** | **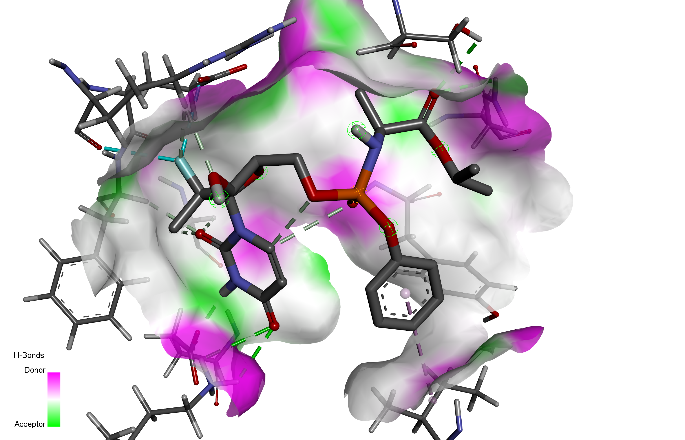** |
| **(E)** | **(F)** |
| 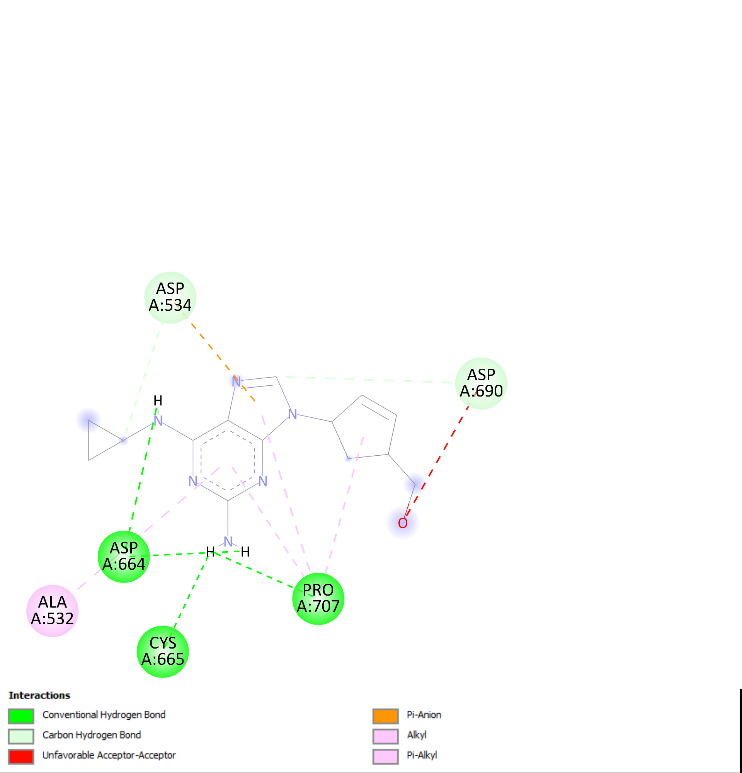 | 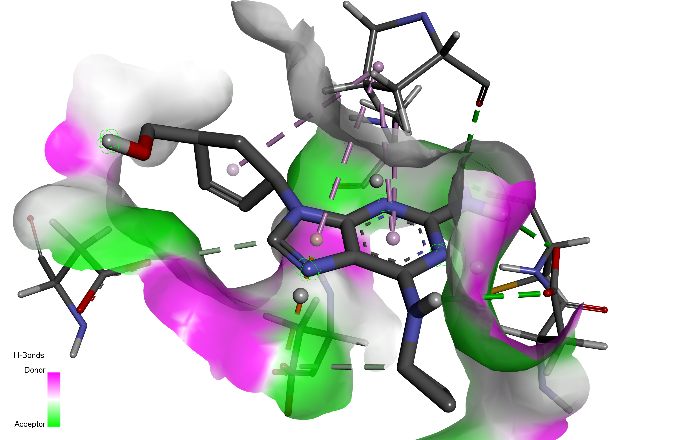 |

Figure S10. Docking of RdRp of Dengue (5K5M) with Remdesivir interaction (A) binding pattern (B), Sofosbuvir interactions (C) binding pattern (D), and Abacavir interactions (E) binding pattern (F).

| **(A)** | **(B)** |
| --- | --- |
| **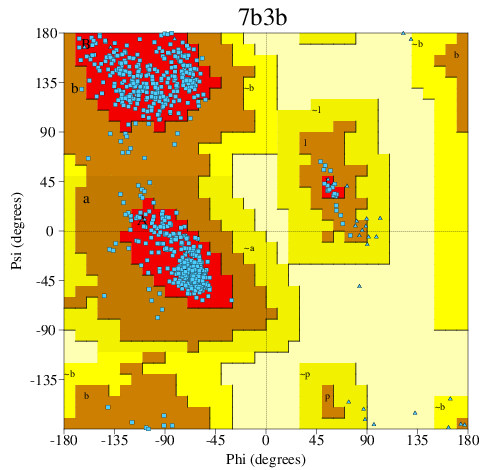** | **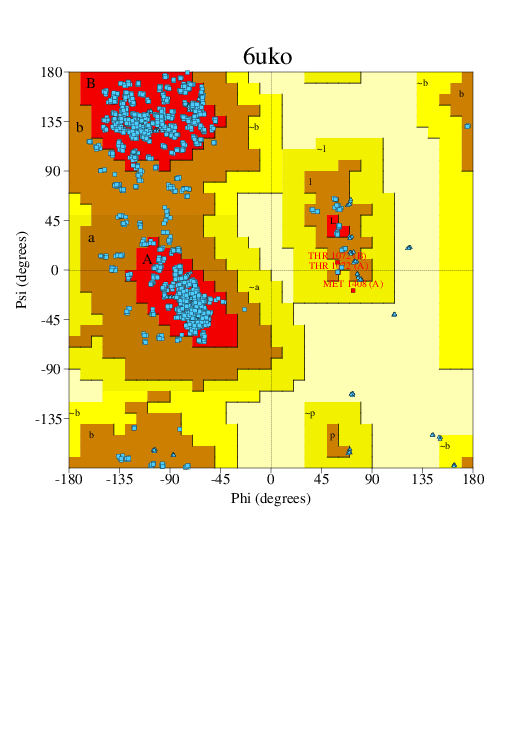** |
| **(C)** | **(D)** |
| 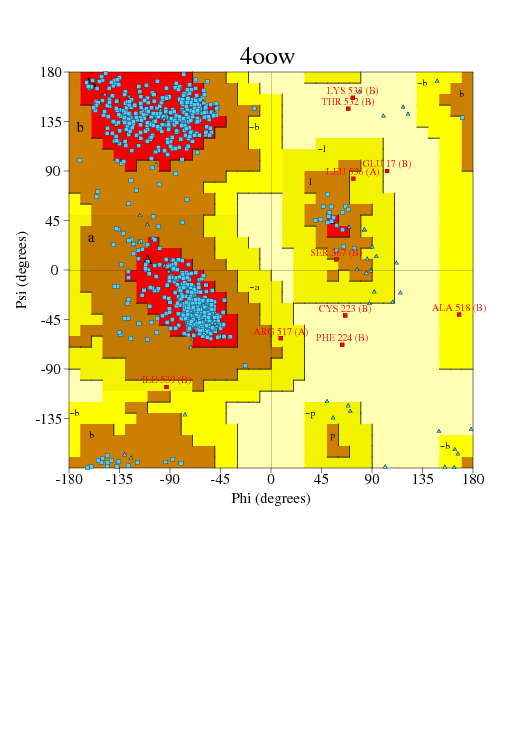 | 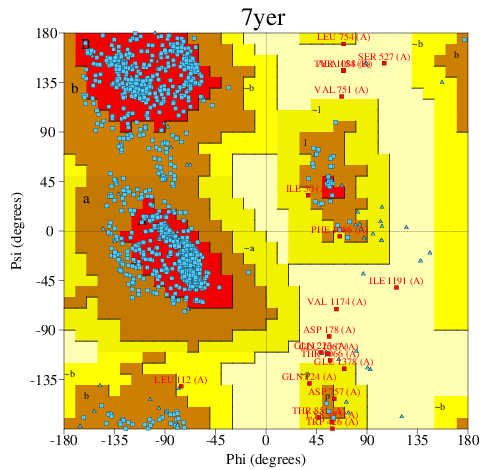 |
| **(E)** | |
| 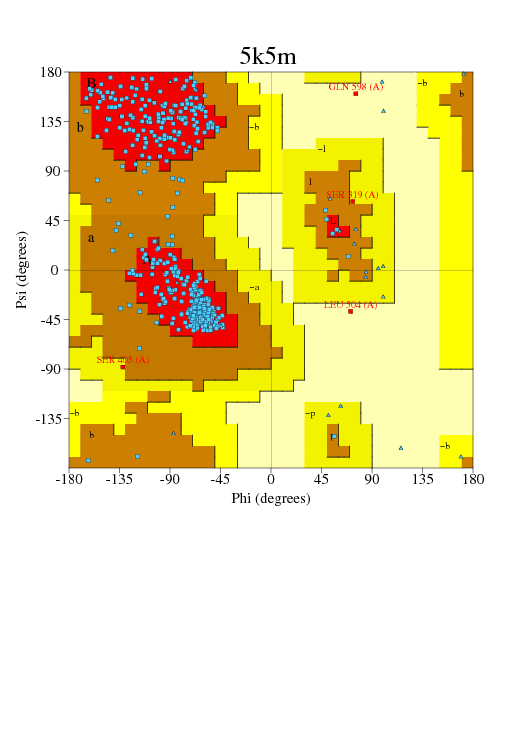 | |

Figure S11. Ramachandran plot of RdRp receptor protein structures SARS-CoV-2 (A), HIV-1 (B), Hepatitis C (C), Ebola (D), and Dengue (E) analyzed by PROCHECK online web tool.

| **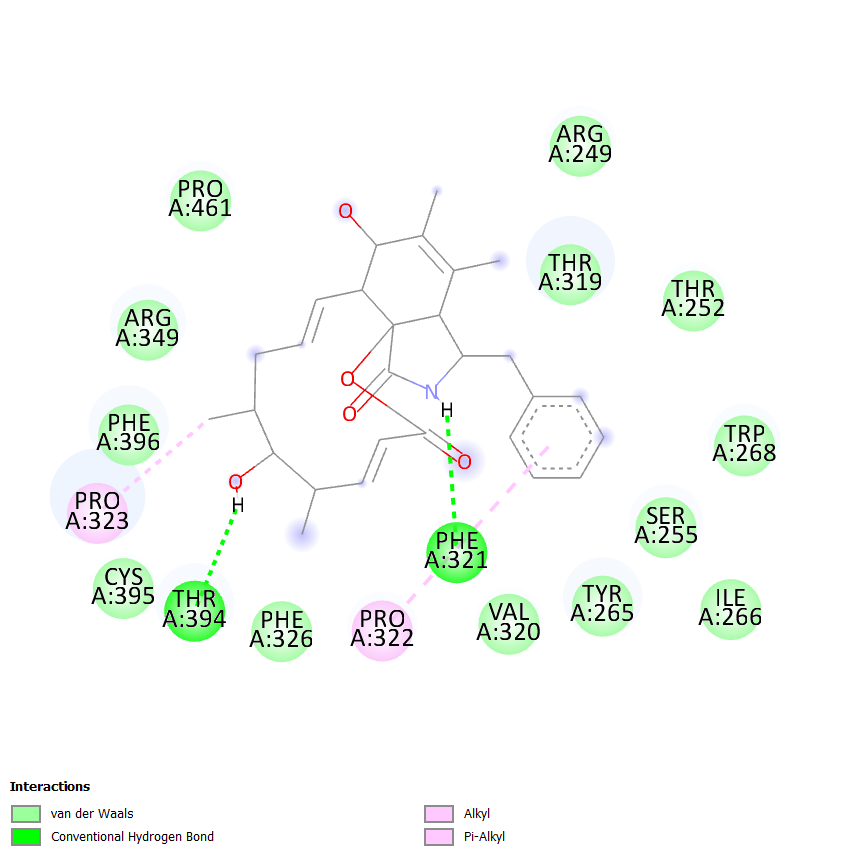** | **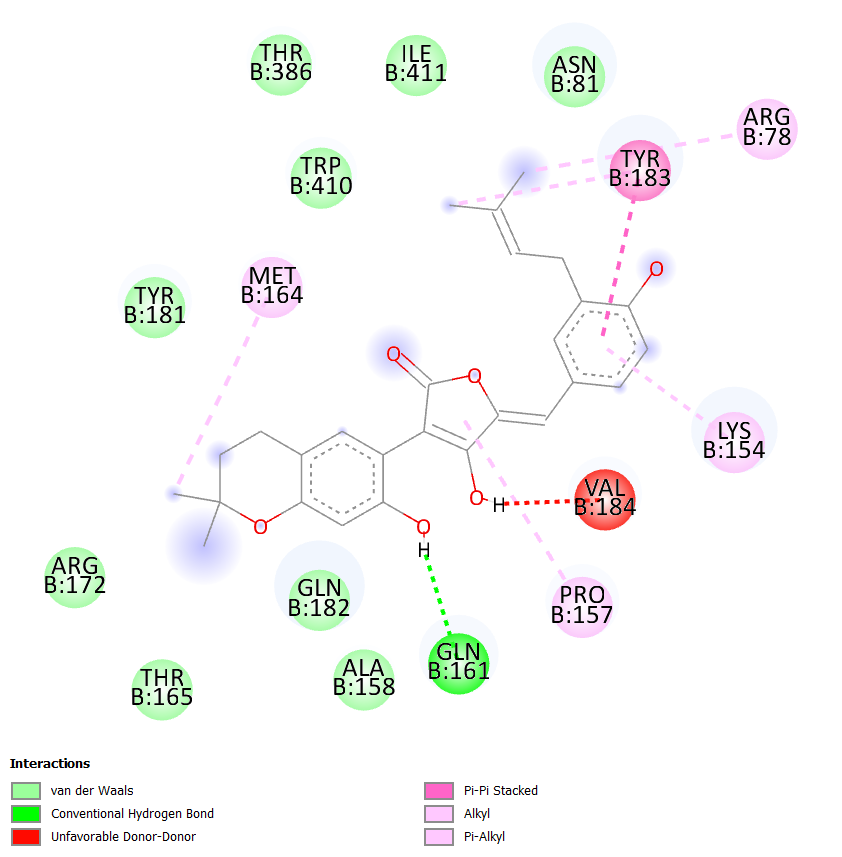** |
| --- | --- |
| **(a)** | **(b)** |
| **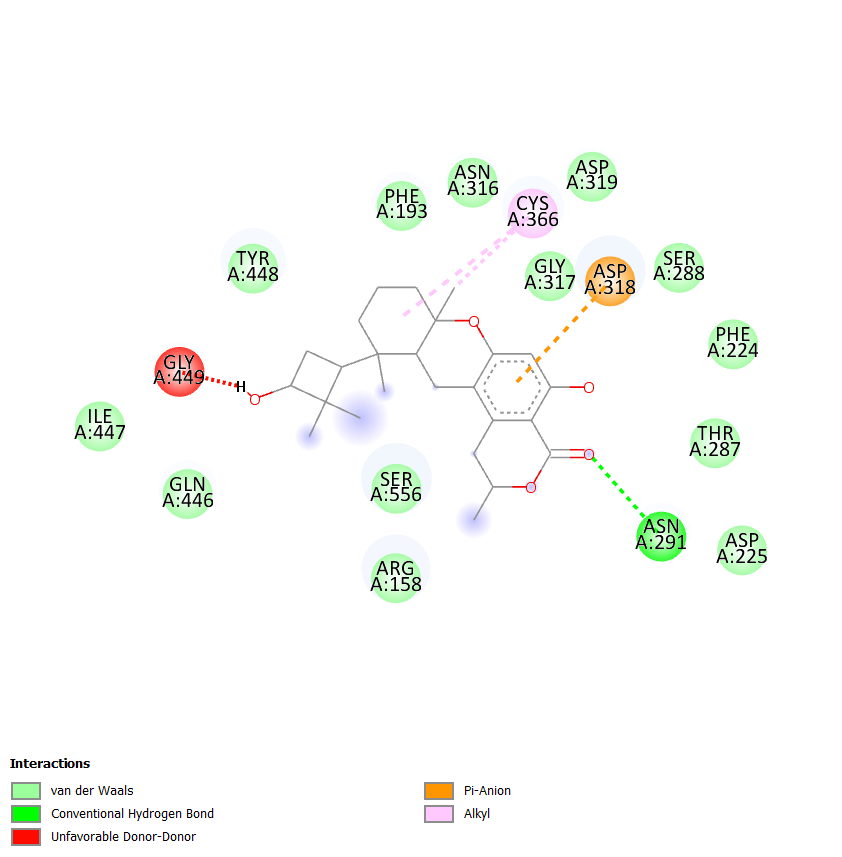** | **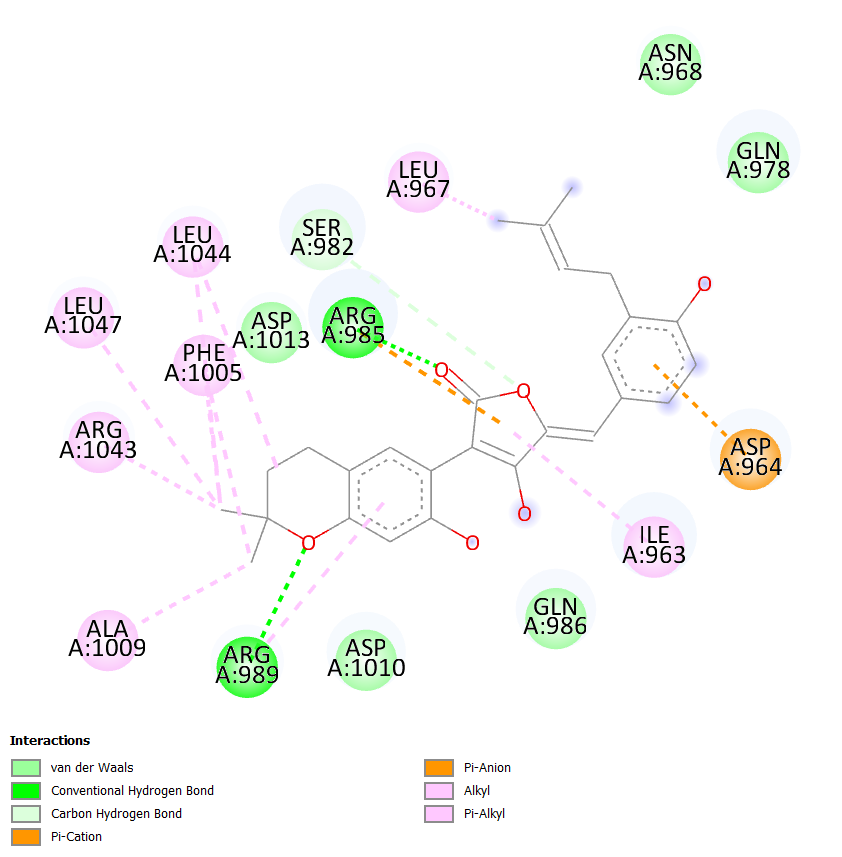** |
| **(c)** | **(d)** |
| **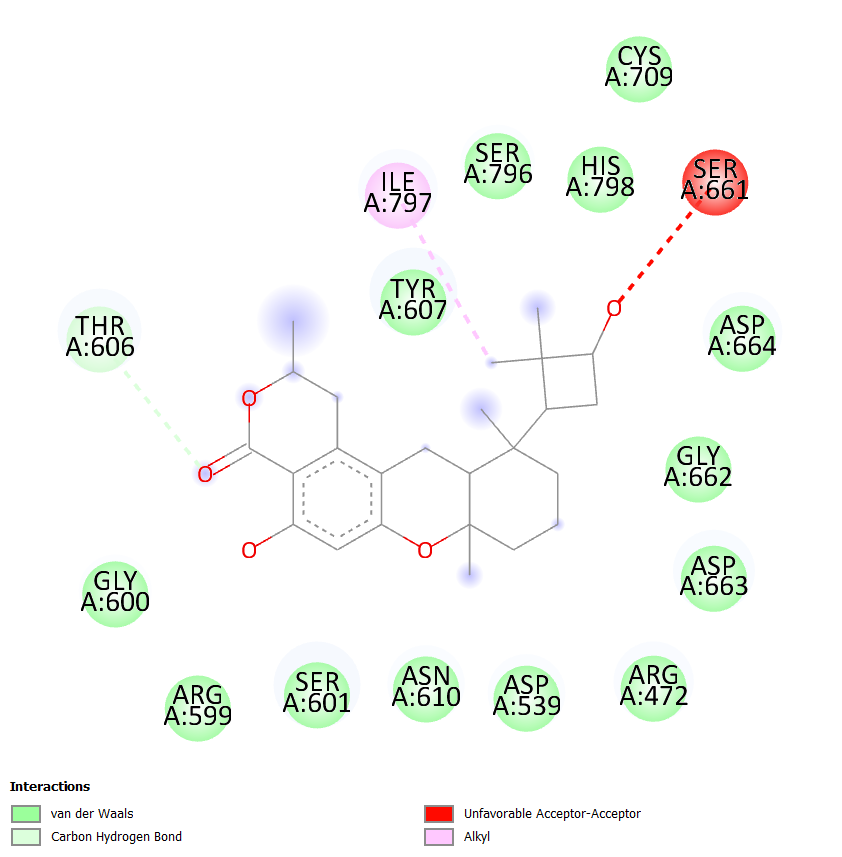** | **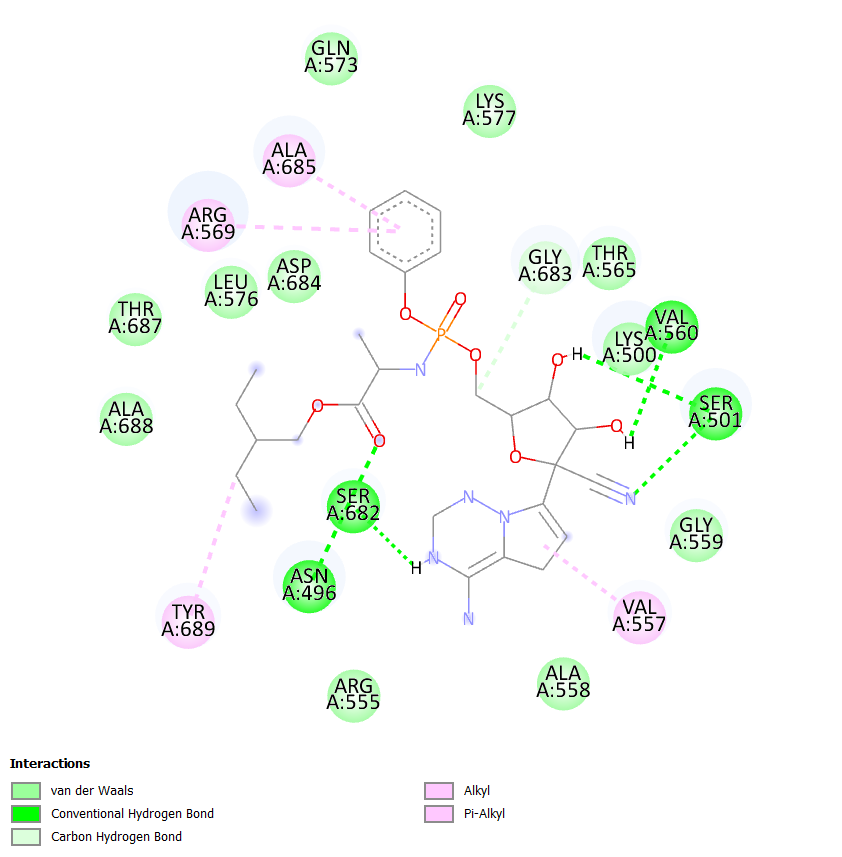** |
| **(e)** | **(f)** |

Figure S12. First pose 2D images of MD trajectories of the best selected ligands and remdesivir as a control drug. (a) RdRp of SARS-CoV-2 and cytochalasin Z8 complex, (b) RdRp of HIV-1 and aspulvinone D complex, (c) RdRp of hepatitis C and talaromyolide D complex, (d) RdRp of Ebola and aspulvinone D complex, (e) RdRp of dengue and talaromyolide D complex, (f) RdRp of SARS-CoV-2 and remdesivir complex.

| **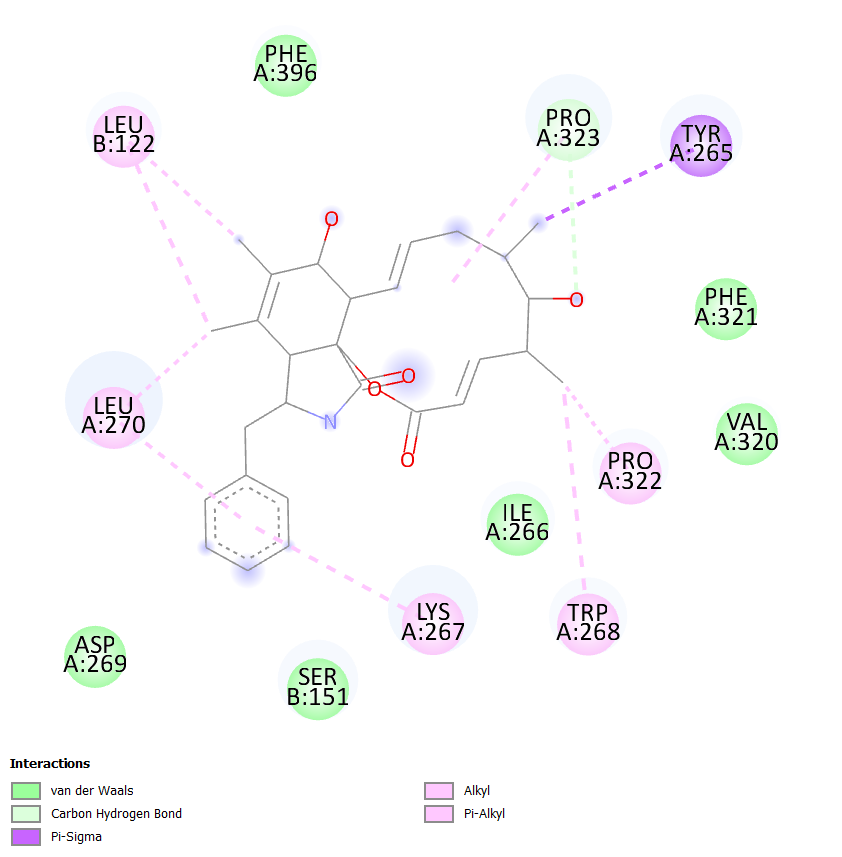** | **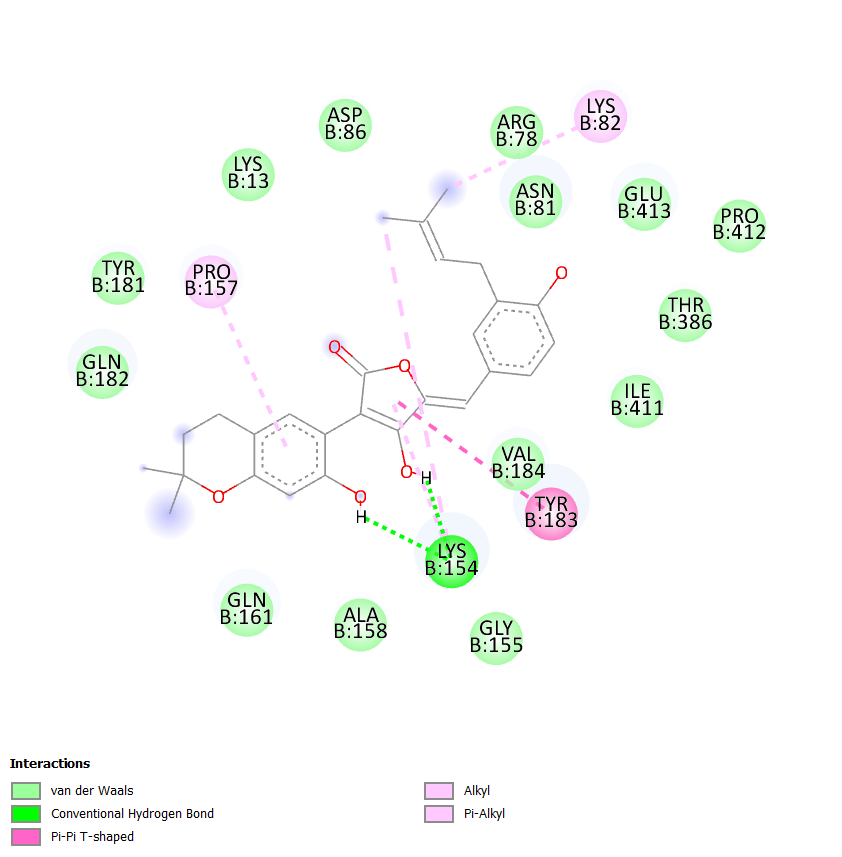** |
| --- | --- |
| **(a)** | **(b)** |
| **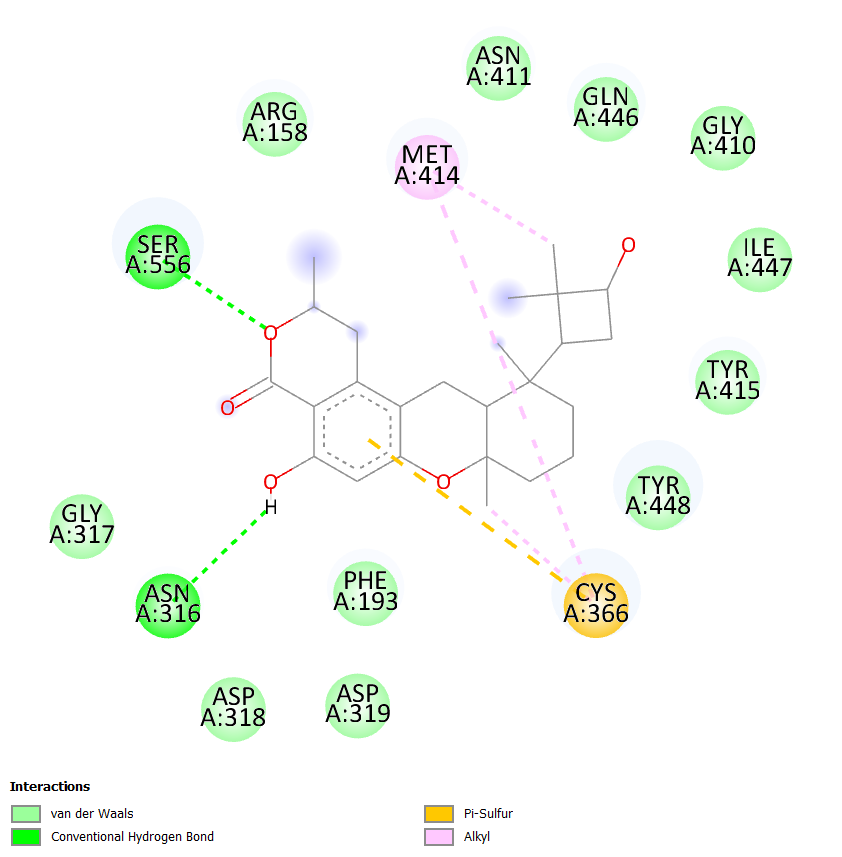** | **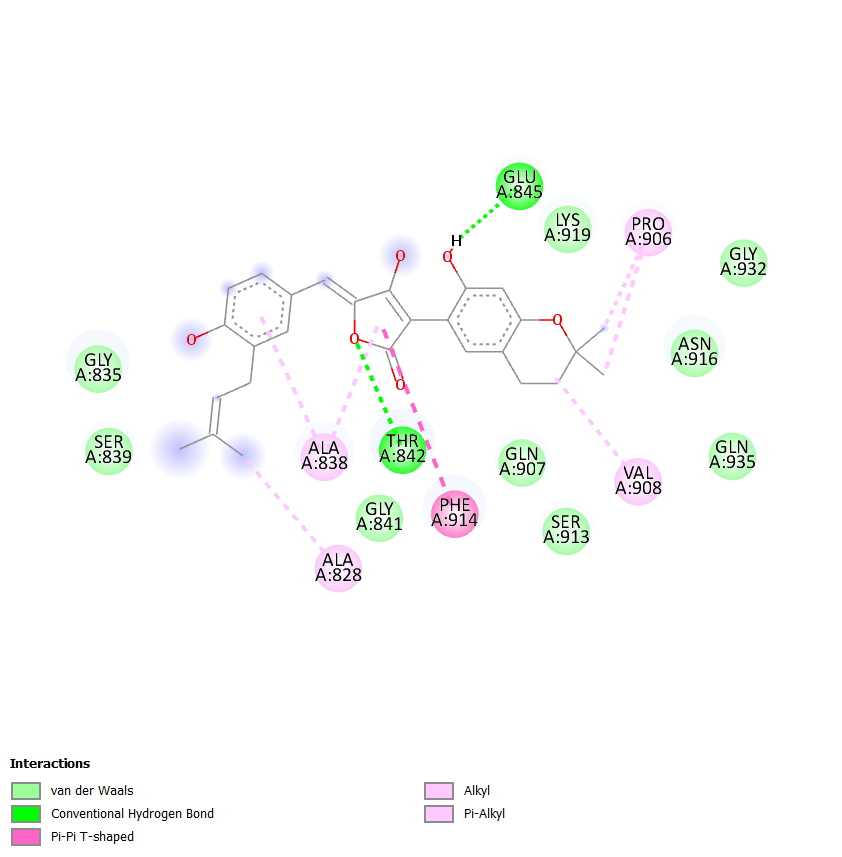** |
| **(c)** | **(d)** |
| **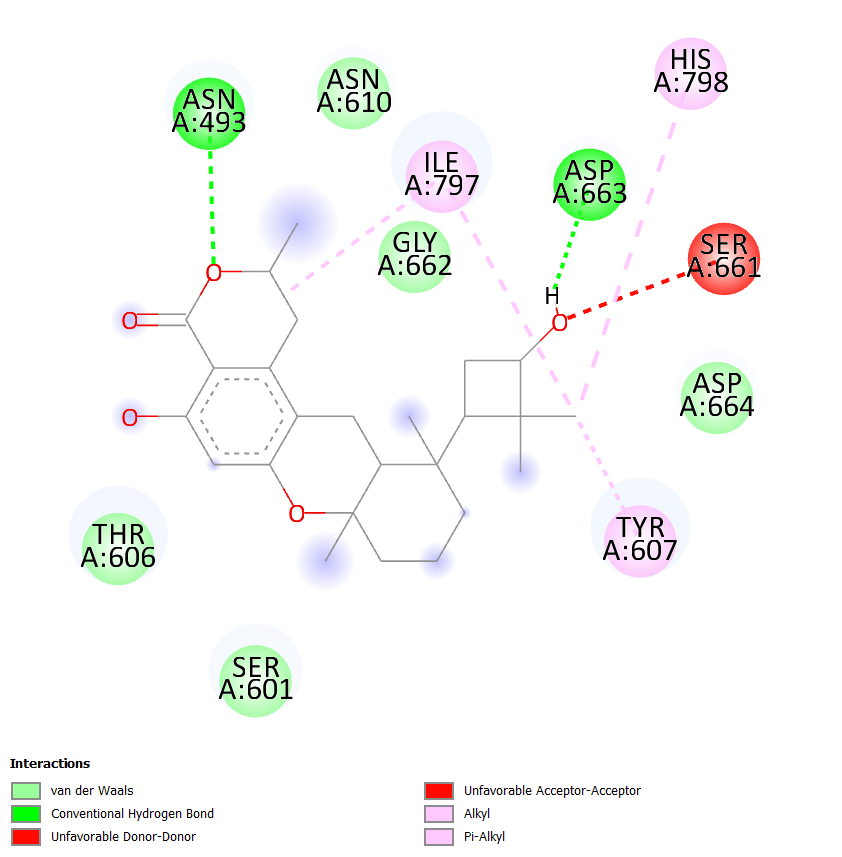** | **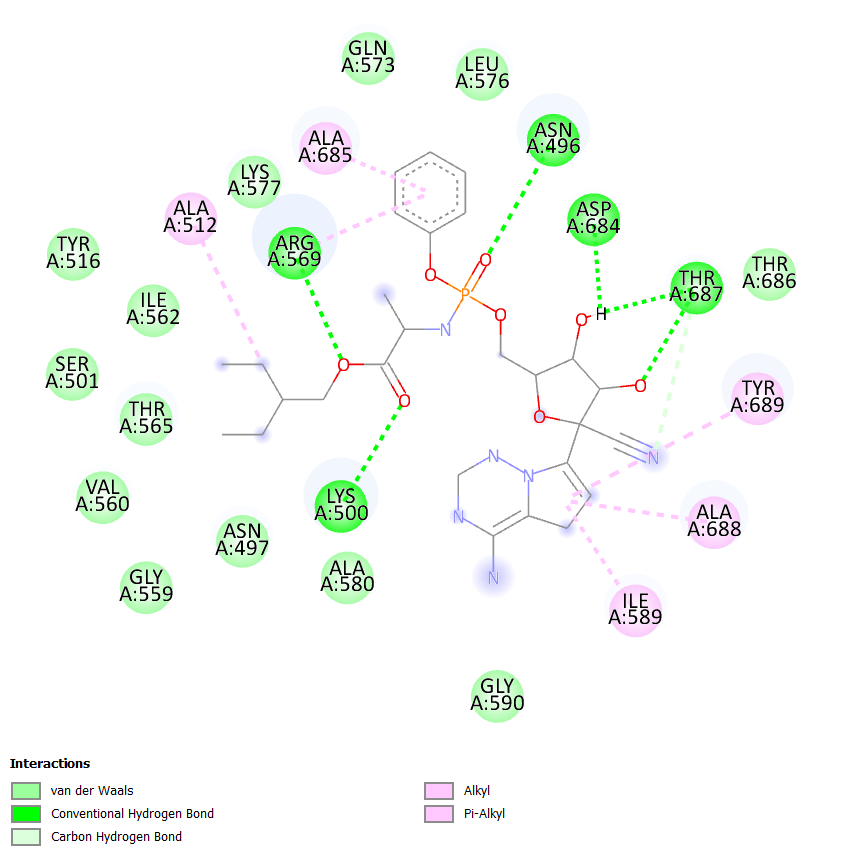** |
| **(e)** | **(f)** |

Figure S13. Last pose 2D images of MD trajectories of the best selected ligands and remdesivir as a control drug. (a) RdRp of SARS-CoV-2 and cytochalasin Z8 complex, (b) RdRp of HIV-1 and aspulvinone D complex, (c) RdRp of hepatitis C and talaromyolide D complex, (d) RdRp of Ebola and aspulvinone D complex, (e) RdRp of dengue and talaromyolide D complex, (f) RdRp of SARS-CoV-2 and remdesivir complex.
